# Framework for multi-criteria assessment of classification models for the purposes of credit scoring

**DOI:** 10.1186/s40537-023-00768-7

**Published:** 2023-06-02

**Authors:** Paweł Ziemba, Jarosław Becker, Aneta Becker, Aleksandra Radomska-Zalas

**Affiliations:** 1 Institute of Management, University of Szczecin, Szczecin, Poland; 2 Faculty of Technology, The Jacob of Paradies University, Gorzów Wielkopolski, Poland; 3 Faculty of Economics, West Pomeranian University of Technology, Szczecin, Poland

**Keywords:** Classification algorithms, Model evaluation, Multi-criteria decision making, PROSA, PROMETHEE II, Credit scoring

## Abstract

The main dilemma in the case of classification tasks is to find—from among many combinations of methods, techniques and values of their parameters—such a structure of the classifier model that could achieve the best accuracy and efficiency. The aim of the article is to develop and practically verify a framework for multi-criteria evaluation of classification models for the purposes of credit scoring. The framework is based on the Multi-Criteria Decision Making (MCDM) method called PROSA (PROMETHEE for Sustainability Analysis), which brought added value to the modelling process, allowing the assessment of classifiers to include the consistency of the results obtained on the training set and the validation set, and the consistency of the classification results obtained for the data acquired in different time periods. The study considered two aggregation scenarios of TSC (Time periods, Sub-criteria, Criteria) and SCT (Sub-criteria, Criteria, Time periods), in which very similar results were obtained for the evaluation of classification models. The leading positions in the ranking were taken by borrower classification models using logistic regression and a small number of predictive variables. The obtained rankings were compared to the assessments of the expert team, which turned out to be very similar.

## Introduction

The COVID-19 pandemic and related panic and restrictions have had a huge, negative impact on the global economy. The decline in potential labour income lowered consumer demand, and many business sectors either closed down or experienced financial difficulties [1]. The economic crisis caused by the pandemic is considered to be several times bigger than the global financial crisis of 2007–2009 [2]. In times of crisis, financial institutions, e.g. banks, have to limit the occurrence of risk in their activities [3]. In practice, the main types of risk that commercial banks face today are credit risk, interest rate risk and operational risk [4]. These risks are interrelated, e.g. as interest rates increase, the risk of floating interest rate loans increases. Of the above-mentioned risks, the main one is credit risk, which determines whether the borrower is able to repay the loan on time. Therefore, research on commercial banks’ credit risk is of significant theoretical and practical importance [4]. An important aspect in this context is the distinction between credit risk and the bank’s proficiency at evaluating credit risk and monitoring the loans it has made [5]. Banks use the so-called Credit Scoring Systems, which, based on the collected data about customers, conduct a credit risk analysis in order to make a final credit decision [6]. Credit risk assessment is most often performed on the basis of historical data [7] with the use of classification methods constituting the basis for the construction of classification models for the purposes of credit scoring [8].

The main dilemma in the case of classification tasks is the selection of an appropriate algorithm adapter to the problem under consideration [9]. The formalized description of the algorithm selection problem proposed in 1976 by Rice [10] takes the form of abstract 5-element models composed of performance measures and the problem space, algorithms, features and criteria. Wolpert and Macready [11] claim that there is no single algorithm that could achieve the best performance for all measures in a given problem domain. The results of classification algorithms must be carefully assessed and analysed, and this analysis must be correctly interpreted for further evaluation [12]. Empirical evaluation is the basis for verifying the potential of classification algorithms and models [13, 14].

Therefore, it seems that the proposed ranking of classification algorithms is a better approach to solving a specific classification problem than searching for one algorithm that meets all expectations [15]. According to Peng et al. [16], due to the fact that the ranking of classification algorithms requires the examination of several criteria, e.g. accuracy and precision, the choice of algorithm can be modelled as a multi-criteria decision problem. Classification models are built on the basis of classification algorithms, which are specific products of individual algorithms [17]. Accurate evaluation of classification models is one of the most important parts of the classification process [18], and the ranking of classification models, similarly to the ranking of algorithms, is also a multi-criteria problem. Multi-Criteria Decision Making (MCDM) methods are used in multi-criteria problems of evaluation and ranking of classification models.

MCDM methods are the basis for building decision models, just like classification algorithms are the basis for building classification models. In the case of MCDM methods, it was noted that decision-makers need to understand the method used [19]. Unfortunately, usually the decision maker is not an expert in the field of MCDM methods and has a limited understanding of a given method [20]. As a result, he treats a given method as a ‘black-box’, and this means that he does not trust the results of the MCDM method [21], and may even feel manipulated by the method [20]. In such a situation, it is a big challenge to increase the decision-maker’s confidence in the MCDM method used and the decisions it recommends. The way to increase trust is to align the decision-making model and the decision-maker’s mental model [22]. In addition, the combination of domain expertise and a decision model provides better and more robust decision support [23]. Decision models approximate the empirical reality, but they can also help decision makers understand the implications of their own assumptions and mental models [24]. Therefore, it is important that the decision and mental models are matched, and as a result of this matching, the expert empirical ranking should be consistent with the ranking generated by the decision model.

When it comes to the construction of the ranking of classification models, an important problem that needs to be considered in the assessment of such models is the risk of over-fitting. Over-fitting occurs when the model works well on the training set, but does not cope well with the classification of new cases, e.g. included in the validation set [25]. In practice, it is important to prevent over-fitting, so that the classification model classifies the cases in the training set and in the validation set equally well. Another important problem related to the assessment of credit scoring classification models is the fact that credit scoring prediction is carried out in a changing environment [26]. Therefore, there is a risk of degradation of the performance of the classification model (drift) over time [27]. Moreover, the temporal increase in model error may not be the only sign of its degradation. Some classification models may perform quite well “on average”, but the variability of their error values may fluctuate significantly over time [28]. Error variability degradation is a major challenge for classification models, so it is important that the model has a low variability of classification results over time.

The purpose of the research and the method of its implementation were adopted taking into account all the above-mentioned issues regarding:

- Building a ranking of credit scoring classification models, including empirical evaluation and multi-criteria evaluation,
- Decision-makers’ lack of trust in MCDM methods they are unfamiliar with and the need to increase this trust by matching the results of the decision-making model and the decision-maker’s mental model,
- Risks of over-fitting, drift over time and degradation of the volatility of errors in the credit scoring classification model.

The aim of the research is to develop and practically verify a framework for multi-criteria assessment of classification models for the purposes of credit scoring. The framework takes into account the preferences of the analyst and the future user of the model and supports the expert in choosing the best model from among many variants of models intended for prediction of loan repayment. In this context, it is important to maintain the comparability of the obtained results for different models and to obtain a result in the form of a ranking of classification models as similar as possible to an expert empirical ranking based on a mental model. The framework is based on the MCDM method called PROSA (PROMETHEE for Sustainability Analysis) [29], thanks to which the comparability of individual classification models was ensured. Basing the framework on the PROSA method brings added value to the modelling process, allowing for the evaluation of classifiers to include (1) the consistence of the results obtained on the training set and the validation set, and (2) the consistency of the classification results for the data obtained in different time periods.

The article consists of 6 sections, the first of which is this introduction. The second section presents a review of the literature on the problems of credit scoring, assessment of classification models, including the multi-criteria assessment of classifiers. The third section, materials and methods, contains descriptions of the methods, data and methodological framework used. The fourth section presents the results of the classification models assessment, and the next section presents the discussion, in which the parameters of the assessment model were adjusted in such a way that its results were consistent with the results of the empirical assessment of experts. The article ends with the conclusions.

## Literature review

When selecting algorithms for classification methods, the most common is the conventional approach, which includes, among others, knowledge from experts, trial and error method or theoretical analysis of the issues under consideration. Such proposals, according to Wang et al. [30], however, have the following disadvantages: high computational costs in the case of quite large data sets, inability to obtain knowledge about all classifiers resulting from the assessment of their representational errors, and despite the possibility of cooperation with field experts, such a solution also requires significant financial and correct relations with specialists. At the same time, Khan et al. [31] indicated that there is a noticeable increase in demand for machine learning systems that could automate the process of selecting appropriate algorithms by recommending them for various tasks. In their opinion, such systems do not have the disadvantages of conventional approaches and allow the use of machine learning algorithms to solve new problems, and also allow non-experts to operate independently.

### Credit scoring predictive models

Credit risk assessment is important for financial institutions, companies and regulators. Its result is influenced, among others, by skilful risk management, identification and understanding of the factors on which it depends. On the other hand, scoring systems are important tools used to assess and monitor credit risk. Providing the most accurate risk forecast is the most important task for scoring models. The additional expectations of regulatory authorities that these models should be transparent and auditable means that simple predictive models, such as logistic regression or decision trees, are still used in practice today. Another proposed approach in the literature is the use of a wider spectrum of machine learning models, although according to Bücker et al. [32] their predictive potential is not fully exploited, leading to higher provisions or more outstanding loans. Dastile et al. [33] noted that despite the advanced applications of machine learning models in credit scoring, there are two fundamental problems: the incapability of some of the machine learning models to explain predictions and the issue of imbalanced datasets. The authors reviewed the literature describing the use of statistical approaches, machine learning and deep learning in credit scoring, identified existing limitations, leading and emerging directions in this field. According to Dastile et al. [33], the group of classifiers outperforms single classifiers, while deep learning models (e.g. convolutional neural networks) showed better results compared to other models. In the literature on credit scoring, explanatory data analysis, the role of macroeconomic variables (e.g. interest rates, unemployment and inflation) and the study of the correlation relationship between variables are often overlooked.

Among the recently published studies, the work of Trivedi [34] deserves attention, which focused on building a predictive credit scoring model taking into account German credit data. According to the author, who conducted a series of comparative analyses, the use of different feature selection techniques (such as Information-gain, Gain-Ratio and Chi-Square) and machine learning classifiers (Bayesian, Naïve Bayes, Random Forest, Decision Tree (C5.0) and SVM (support Vector Machine) contributed to improving the prediction of credit scoring. The work of Teles et al. [35] presents a comparison of research results obtained using fuzzy sets with decision trees based on artificial neural networks on credit scoring to predict the recovered value. The authors pointed out that both models allow modelling uncertainty. However, fuzzy logic is more accurate in this respect, despite the difficulties with its implementation. On the other hand, presenting the problem itself is more beneficial in the case of using a decision tree. According to Kumar and Gunjan [36], machine learning is offering immense potential in Fintech space and determining a personal credit score, and entities using deep learning and machine learning techniques have the ability to serve people who do not use the services of traditional financial institutions. The test analyses of the proposed machine learning model carried out by the authors showed that it is effective and allows for a better analysis process compared to solutions not related to machine learning.

A significant number of machine learning models have been used by Provenzano et al. [37] to create a state-of-the-art. credit scoring and default prediction system. In the presented research, the authors used the latest ML/AI concepts, starting with natural language processes (NLP) applied to (textual) descriptions of economic sectors using embedding and autoencoders (AE), followed by the classification of insolvent companies using gradient boosting machines (GBM) and calibrating their probabilities, then assigned credit ratings using differential evolution DE). The interpretability of the model was achieved by implementing techniques such as SHAP and LIME, which explain predictions locally in features’ space.

An important indicator for investors and decision-makers that should be taken into account in credit scoring work is the index of economic freedom, which enables the assessment of the degree of market openness over the degree of fiscal and regulatory restrictions. The work of Puška et al. [38] presented a multi-criteria ranking of the Balkan countries based on the criteria of economic freedom. The weight of the criteria was determined using the Entropy method, and the countries were ranged using the CRADIS method (Compromise Ranking of Alternatives from Distance to ideal Solution) using a double normalisation approach, which, according to the authors, contributed to the stability of decision-making.

According to Doumpos and Zopounidis [39], multi-criteria decision (MCDA) provides analytical methodological tools for decision support based on multiple conflicting criteria and is suitable for financial decision support. MCDA participates at all levels of the financial decision-making process. It includes the stages of problem structuring and algorithmic issues related to constructing and evaluating satisfactory solutions. Roy and Shaw [40] drew attention to the few studies on sustainability credit score systems (SCSS). The authors proposed a multi-criteria SCSS, which took into account financial and management as well as environmental and social aspects. They used a combination of the Best–Worst Method (BWM) and the fuzzy-Technique for Order Preferences by Similarity to an Ideal Solution (TOPSIS) method to create a credit scoring system. BWM was used to weight factors and fuzzy-TOPSIS was used to evaluate candidates. According to the authors, the obtained solutions will help financial institutions identify borrowers who engage in sustainable business practices. Noteworthy is the proposal of the hybrid MCDM method on the Pythagorean fuzzy-environment discussed in the work by Chaurasiya and ain [41]. According to the authors, the proposed approach can be used to identify the best software used for efficient banking management software (BMS). This method is based on the Pythagorean Fuzzy Method based on Removal Effects of Criterion (PF-MEREC) and Stepwise Weight Assessment Ratio Analysis (SWARA) approaches. The objective and subjective weights are assessed by PF-MEREC, SWARA model and the preference order ranking of the various alternatives is done through Complex Proportional Assessment (COPRAS) framework on the PFS.

### Issues choosing the right model and classification algorithm

According to Kalousis and Theoharis [42], the selection of an appropriate classification model and algorithm is essential for the effective discovery of knowledge on a data set. As factors that make the selection task difficult, the authors listed many criteria of classifiers’ performance and the features of the data set affecting this performance. They proposed the use of an intelligent assistant (NOEMON), which supports the selection of an appropriate classifiers. Khan et al. [31] emphasize that classification is the key and most studied paradigm in the machine learning community. However, choosing the right classification algorithm that can be used to solve a specific problem is quite a difficult task. The mentioned dilemma is formally referred to in the literature as the algorithm selection problem (ASP). The authors’ work presents a comparative assessment of, in their opinion, all known methods of selecting classifiers, based on 17 classification algorithms and 84 sets of comparative data, as well as conclusions and recommendations. According to Brodley [43], the results of empirical comparisons of learning algorithms show that each algorithm has a selective superiority. This means that it is best for some but not all tasks. Due to the dataset, it is often impossible to say a priori which algorithm will provide the best performance. For some tasks, it is reasonable to use different classifiers, and then it is suggested to create a hybrid classifier that will include the best properties of individual algorithms. Whereas Amancio et al. [44] argue that in works on classifiers, the research focuses primarily on the performance of a given algorithm or the comparison of different classification methods. In many cases, in their opinion, researchers who are not machine learning experts struggle with practical classification tasks without adequate knowledge of the underlying parameters and use their default configuration. As a result of their experiments, the researchers noticed that there is a strong influence of the number of features on the performance of classifiers and that there are different responses of algorithms to the same set of variables. In turn, Vela et al. [28] found that the time dependence of the classification model results was practically ignored in classifier implementations. They noted that it is generally accepted that once a model has been trained to the required quality, it is ready to be deployed and used without further updating or retraining. However, data-generating environments often change over time, and their statistical properties change with them. This data evolution, known as “concept drift”, inevitably affects the quality of the models to the point where the model may no longer correspond to the new reality.

In the literature on the subject, there are many proposals and applications of classifiers in various fields. Interesting research results were published by Y. Wu et al. [45]. They assess the ability of four machine learning classifiers (i.e. multinomial logistic regression—MLR; support vector machine,—SVM; random forest—RF; gradient boosting trees—GBT) for mapping lake ice cover, water and cloud cover during both break-up and freeze-up periods using the MODIS/Terra L1B TOA (MOD02) product. Accuracy assessment using random k-fold cross-validation (k = 100) showed that all machine learning classifiers using a 7-band combination (visible, near infrared, and shortwave infrared) are able to achieve an overall classification accuracy greater than 94%. According to the authors, only RF was relatively insensitive to the choice of hyperparameters compared to the other three classifiers, demonstrating the potential of RF to map lake ice cover around the world based on the reflection data from MODIS TOA. In the publication on land-use/land-cover change (LULC), Talukdar et al. [46] presented a quantified assessment of these changes. They highlighted the need to investigate the accuracy of various LULC mapping algorithms to identify the best classifier needed to conduct further earth observations. The research involved six machine learning algorithms: random forest (RF), SVM, ANN, Fuzzy ARTMAP, SAM and the Mahalanobis distance (MD). Accuracy was assessed using the Kappa coefficient, ROC curve, index-based validation and root mean square error (RMSE). The results of the Kappa coefficient indicated that the applied classifiers had a similar level of accuracy, with the RF algorithm having the highest and, according to the authors, the best ML classifier, while the MD algorithm had the lowest accuracy. The main goal of the study by J. Roy and S. Sah [47] was to assess the vulnerability to erosion of the gorge (Hinglo river basin, an important tributary of the Ajay river—India), which combined approaches based on artificial intelligence and machine learning. A multi-layer perceptron network (MLP) was used as the base classifier, and hybrid machine learning methods, i.e. Bagging and Dagging, were used as functional classifiers. The ROC curves, mean absolute errors (MAE) and root mean square error (RMSE) were used to evaluate and compare the models. According to the authors, the integration of hybrid models with MLP increased the accuracy of the MLP models. The highest accuracy was achieved by MLP-Dagging.

The aim of the research presented by Kartal et al. [48] was to develop a hybrid methodology integrating machine learning algorithms with MCDM methods to efficiently perform multi-attribute inventory analysis. The appropriate class for each inventory item was determined on the basis of the results of the ABC (Activity Based Costing) analysis using three MCDM methods, i.e.: SAW (Simple Additive Weighting), AHP (Analytical Hierarchical Process), VIKOR (from Serbian: VIseKriterijumska Optimizacija I Kompromisno Resenje, that means: Multicriteria Optimization and Compromise Solution). In the next step, the naïve Bayesian, Bayesian network, artificial neural network (ANN) and support vector machine (SVM) algorithms were implemented to forecast the classes of predefined inventory items. Final activities focused on determining the detailed prediction performance metric of the algorithms for each method. The authors indicated that SSN and SVM are precise classifiers, both of which can be effectively applied to the issue of inventory management in a multi-criteria approach.

The efficiency of supervised classifiers was analysed in connection with the classification of biomedical data by Tuysuzoglu and Yaslan [49]. According to the researchers, the development of information technology has contributed to the improvement of storage and analysis of biomedical data sets, while machine learning methods have made a significant contribution to the evaluation and interpretation of this data. The authors obtained the optimal results of classification accuracy using SVM (Support Vector Machines) and Dictionary Learning methods, RDL (Random Feature Subspaces) and BDL (Random Instance Subspaces), which are generated using random feature/instance subspaces. Chauhan and Singh [50] proposed the use of machine learning in the diagnosis of cervical cancer to detect malignant neoplastic cells in the initial stage. They noted problems with data imbalance and non-uniform scaling across the dataset. That’s why they used Synthetic Minority Oversampling Technique along with fivefold cross-validation. The authors compared the performance of popular machine learning (ML) classifiers, such as: Naive Bayes, Logistic Regression, K-Nearest Neighbor, Support Vector Machine (SVM), Linear Discriminant Analysis, Multi-Layer Perceptron, Decision tree (DT) and Random Forest (RF) on unscaled and scaled data obtained by applying: Min–Max scaling, standard scaling and normalization. The authors proposed the best three ML algorithms in the discussed problem: RF, SVM and DT. The optimization possibilities were investigated with the methods of feature selection: univariate feature selection and recursive feature elimination (RFE). The best overall performance was obtained with the RFE random forest (RF-RFE). According to Chand et al. [51], Support Vector Machine (SVM) is one of the better classification algorithms specifically used to detect network intrusions. The authors indicated that it should be combined with other classifiers to improve performance. Research in this area has shown that the integration of SVM and random forest is an algorithm with a better classification power, especially for detecting low-frequency attacks, such as password guessing or spyware detection.

Ji et al. [52] believe that advances in machine learning have led to the increased deployment of black-box classifiers in many different applications. According to the authors, the performance of these pre-trained models should be critically and reliably assessed. Therefore, they presented an active Bayesian approach to assess the classifier performance. To this end, they performed a series of systematic empirical experiments evaluating the performance of modern neural classifiers (e.g. ResNet and BERT) on several standard image and text classification data sets. On the other hand, Gu and Jin [53] proposed an innovative partially supervised team learning algorithm called Multi-Train. It generates a number of heterogenous classifiers that use different classification models and/or different characteristics. According to the authors, the use of various input models and functions improves the performance of the presented approach compared to the existing supervised classifiers.

### Overview of MCDM applications in the assessment and selection of classifiers

Many authors of publications that have appeared in recent years indicate and argue that MCDM methods are practical tools useful in the selection of machine learning (ML) classification algorithms. However, individual methods in their assessment can focus on different properties of classifiers, which results in obtaining divergent rankings. Therefore, it is often postulated to integrate several techniques, which will result in the development of a compromise, final statement. This section reviews the applications of MCDM methods for the assessment and selection of classifiers, the results are summarized in Table 1.

**Table 1:** Overview of the applications of the MCDM methods for the assessment and selection of classifiers

Purpose and subject of the study	No. of classifiers (alternatives)	No. of criteria	Data sets	Applied MCDM methods	Refs.
Use of a set of MCDM methods to evaluate classification algorithms for software defect detection	38	13	10 public-domain software defect datasets	DEA, TOPSIS, ELECTRE and PROMETHEE II	[16]
An approach to resolve disagreements among MCDM methods based on Spearman’s rank correlation coefficient	17	10	over 11 public-domain binary classification datasets	TOPSIS, ELECTRE, GRA, VIKOR, PROMETHEE	[54]
The choice of classification algorithm in Machine Learning	7	10	Australian public domain credit data set	FAHP, TOPSIS, SAW	[55]
Finding of a robust classifier, which is suitable for consideration as the base learner, while designing a host-based or network-based intrusion detection system	54	16	the NSLKDD, ISCXIDS2012, CICIDS2017 datasets	TOPSIS	[56]
An accurate multi-criteria decision making methodology (AMD) which empirically evaluates and ranks classifiers’ and allow end users or experts to choose the top ranked classifier for their applications AMD methodology presents an expert group-based criteria selection method	35	4 (selected by experts out of 8 features)	15 publicly available UCI and OpenML datasets	AHP, TOPSIS	[57]
Comparing the performance of algorithms those are used to predict diabetes using data mining techniques	5	3	1 data set from UCI machine learning data repository	comparison of criterion values	[58]
A new classification algorithm recommendation method based on link prediction between data sets and classification algorithms	21	5	131 publicly available UCI data sets	proposition of own method based on: prediction and Data and Algorithm Relationship (DAR) Network	[59]

*MCDM* multi-criteria decision making, *DEA* data envelopment analysis, *TOPSIS* technique or order of preference by similarity to ideal solution, *ELECTRE* from French: ÉLimination et Choix Traduisant la REalité, that means: ELimination Et Choice Translating REality, *PROMETHEE* Preference Ranking Organization METHod for Enrichment of Evaluations, *GRA* grey relational analysis, *VIKOR* from Serbian: VIseKriterijumska Optimizacija I Kompromisno Resenje, that means: Multicriteria Optimization and Compromise Solution, *AHP* analytical hierarchical process, *FAHP* fuzzy analytical hierarchical process, *SAW* simple additive weighting.

According to Kou et al. [54], the MCDM methods are suitable tools for selecting classification algorithms, which is an important issue for many disciplines. The authors proposed a solution based on the Spearman’s rank correlation coefficient. For this purpose, five MCDM method were tested—i.e.: TOPSIS (Technique or Order of Preference by Similarity to Ideal Solution), GRA (Grey Relational Analysis), VIKOR, PROMETHEE II (Preference Ranking Organization METHod for Enrichment of Evaluations) and ELECTRE III (from French: ÉLimination et Choix Traduisant la REalité, that means: ELimination Et Choice Translating REality)—using 17 classification algorithms and 10 performance measures on 11 public domain binary classification data sets, and as a result, consistency was achieved between the analysed multi0criteria methods. Satisfactory results were obtained by determining the weight for each MCDM method in accordance with the similarities between the ranking generated by the method and the rankings generated by the other algorithms. According to Awodele et al. [55], the selection of a classification algorithm is a major problem in Machine Learning (ML) and the algorithm selection process can also be modelled as an MCDM problem. The authors presented research focused on seven classification algorithms and ten performance criteria. The aim of these activities was to test the proposed FAHP (Fuzzy Analytical Hierarchical Process) and TOPSIS (Technique or Order of Preference by Similarity to Ideal Solution) models. FAHP was used to assign weights to criteria and to rank performance criteria, while the SAW (Simple Additive Weighting) and TOPSIS task was to rank the classifiers. The result of the ML algorithms ranking showed that LRN (Logistic Regression) was in the highest position, thus the authors considered it the best classifier. They also pointed out that MCDM techniques can be an effective tool to help choose the best supervised machine learning algorithm.

Panigrahi et al. [56] point out that the literature lacks proposals for measures to assess the classifier’s performance that would take into account the model construction time, misclassification index and precision. Their observations show that the most frequent use of decision trees and function-based approaches in research is a strong focus on accuracy. In their work, the authors analysed fifty-four popular classifiers that they used in the problem of network intrusion detection and thirteen performance indicators. The aim of the research was to recognize a robust classifier, which is suitable for consideration as the base learner, while designing a host-based or network-based intrusion detection system. The obtained ranking of classifiers, acquired by the TOPSIS method, indicated that J48Consolidated is the best classifier for the design of intrusion detection systems (IDS). According to the authors, it provides the highest accuracy, low misclassification rate and high Kappa coefficient.

The work of Ali et al. [57] discusses the Accurate Multi-criteria Decision-making methodology (AMD), by means of which the classification can be assessed. The user or expert, taking into account their preferences, has the possibility to choose the highest rated classifier in order to build classification models with its help. According to the authors, this proposal results from the situation that the available methods of analysing the results and recommendations of existing classifiers have disadvantages, for example, they do not have: a method of selecting appropriate evaluation criteria, a coherent weighing mechanism or an assessment of the usefulness of classifier results. The article introduces the concept of algorithm quality meta-metrics (QMM) to help experts select appropriate evaluation criteria comparing classifiers, estimates consistent relative weights for evaluation metrics using the analytical hierarchy process (AHP), proposes a statistical significance test and the proposed fit function to filter out algorithms that are statistically insignificant in all scoring criteria. In order to rank the algorithms, the relative proximity value of all algorithms to the ideal ranking was calculated using estimated weights based on AHP and the local and global constraints of the scoring criteria. Consequently, the activities were to evaluate the AMD methodology based on a series of experiments on 15 different classification data sets using 35 classification algorithms. According to the authors, the obtained results of the assessment only confirmed the legitimacy of the proposed solution.

Interesting research results were presented by Kandhasamy and Balamurali [58], who focused on comparing the performance of algorithms used to predict diabetes with the use of data mining techniques. Appropriate grouping of diabetic patients required a comparison of machine learning classifiers (J48 Decision Tree, K-Nearest Neighbors, and Random Forest, Support Vector Machines). The performance of the algorithms was measured for the data set before pre-processing (noisy) and after pre-processing and compared for accuracy, sensitivity and specificity. A comparison of the four diabetes prediction models showed that the J48 Decision Tree classifier achieved the highest accuracy. Repeating the study using a pre-processed dataset identified KNN (K-Nearest Neighbors) and Random Forest as the best classifiers. Zhu et al. [59] emphasize the importance of recommending an appropriate classification algorithm for a given classification problem and indicate that it is one of the most difficult problems in the field of data mining. The authors proposed a method for recommending classification algorithms based on predicting relationships between data sets and classification algorithms. This approach uses prediction, Data and Algorithm Relationship Networks (DARs), takes into account the impact of all datasets and uses interactions between datasets, and between datasets and algorithms. The experiments were based on 131 data sets and 21 classification algorithms, and according to the authors, more effective results were obtained in comparison with ML-KNN [60] (k-NN-based multi-label learning algorithm for recommending proper classification algorithm).

In the work of Peng et al. [16], four MCDM methods were used, i.e. DEA (Data Envelopment Analysis), TOPSIS, ELECTRE and PROMETHEE to rank classification algorithms. The obtained results were obtained on the basis of research using 38 classification algorithms and 13 evaluation criteria in 10 software defect detection data sets (public domain from the NASA Metrics Data Program repository). Due to the nature of the methods used, the analyses took into account the preferences of the decision-maker and, during the ranking procedure, user weights were assigned to performance measures. It should be emphasized that the authors used an impressive set of classification algorithms and team learning algorithms. The WEKA system implements classifiers representing five categories:

- Trees: classification And Regression Tree (CART), Naive Bayes tree and C4.5,
- Functions: linear logistic regression, Radial Basis Function (RBF) network, Sequential Minimal Optimization (SMO) and Neural Networks (NN),
- Bayesian classifiers: Bayesian network, Naive Bayes,
- Lazy classifier: K-nearest-neighbor (KNN),
- Rules: decision Table (DT), Repeated Incremental Pruning to Produce Error Reduction (RIPPER) rule induction [16].

They also used four ensemble methods: bagging, boosting, stacking and vote. In the summary of the research, the authors indicated the two most appropriate algorithms to be used in the discussed problem, which came from the group of decision trees, namely CART and C4.5. For MCDM methods, they provided some contradictory results for the selected datasets, but their propositions were consistent with most of the top-rated classification algorithms. Peng et al. believe that TOPSIS and PROMETHEE II may be more suitable than DEA and ELECTRE I for selecting a classifier performing the software defect detection task.

In publications devoted to classification methods algorithms, it is noted that their performance may differ depending on the measures used and the issues studied, and the selection of the appropriate measure is a difficult task and plays an important role in many areas, e.g. artificial intelligence, operations research, machine learning. It seems important to use the right algorithm for the entire range of proposals developed over the years. However, it is not suggested to use only one intentionally chosen algorithm, but a whole set from which the approach that ensures the best final results can be identified.

An interesting proposal are solutions which consist in treating the choice of an algorithm as an MCDM problem and using methods from this area to select the appropriate measure. This allows, for example, to take into account the user’s preferences, which affect the final assessment and modelling of the task, taking into account criteria [16]. The analysis of the literature listed in Table 1 showed that the number of evaluation criteria (quality and efficiency metrics) of classification models in individual studies, depending on the adopted level of aggregation, took the form of a vector consisting of 3 to 16 elements. The most frequently chosen performance measures include: accuracy, Precision, Sensitivity, Specificity, F-Measure, Kappa, MEA, ROC, overall accuracy, train time, test time, and less frequently used: MCC, PRC, Recall and TP, FP, TN, FN Rates. A systematized approach to the selection of such measures, which stands out from other works, was proposed in the article [57]. The authors constructed eight Quality Meta-Metrics (QMM) which are a categorization of 51 metrics for assessing classifiers available in the Weka system. They postulate and confirm on a practical example that the selection of appropriate meta-metrics and evaluation criteria, assigning them weights, satisfying interdependence and explicit global constraints, enforced by the objectives of the end user’s application should be made by a team of experts from various fields. Whereas only those qualities which satisfy the properties of: legibility, operational, exhaustiveness (containing all points of view), monotonicity and non-redundancy should be selected.

Based on the analysis of the works listed in Table 1, two research gaps in the multi-criteria methodology for the assessment of classification models can be identified.

The gap in the construction of a multi-level criteria structure (determining the appropriate aggregation of these levels) taking into account not only a properly selected set of measures of the performance of classification models, but also:

- Volatility of the values of these measures over time (the degree of their granulation, e.g. month, quarter, year),
- The values of these measures obtained for the training and validation sets.

The gap of gaining the trust of experts (decision makers and analysts who are users of the MCDM method in the form of a friendly tool) by ensuring the compliance of the solution with their mental model (e.g. obtaining the consistency of the results of the MCDM approach with reference results, expert heuristics).

In the context of the identified gaps, the goal of the research can be formulated, which includes the development of a multi-criteria assessment procedure for classification models in the form of a framework. This procedure is to take into account the criteria and weights defined by the expert (or experts) and the conditions resulting from the essence of the classification task (e.g. prediction of repayment of bank loans). It is about building a tool that supports and inspires the expert’s confidence in choosing the best classification model from among many model variants, taking into account the identified research gaps and maintaining the comparability of the results obtained in the form of a ranking of these models.

## Materials and methods

### Research context, decision problem and data

The framework for the multi-criteria assessment of binary classification models for the purposes of credit scoring presented in the article was the next, third stage of innovative research on the Intelligent Analytical Platform (IPA), which is currently offered on the Polish market by BD Poland [61]. IPA is an environment that provides comprehensive support for analytical projects, among others: data integration and exploration, extraction of predictive variables, construction, implementation and monitoring of predictive models (including classification). Its main advantages include the functionality that allows you to:

- Construct and validate many different types of predictive models,
- Automatic generation of a scoring card, rating scale and setting a cut-off point,
- Monitoring the statistical strength and stability of variables and predictive models and analysing the quality of calibration of these models [61].

The IPA is designed for organizations interested in using data to automate decision-making processes in areas including, among others, credit risk assessment and sales support.

The authors of the article were subcontractors of research on IPA commissioned by the main contractor BD Poland in the research and development project entitled “Hybrid system for intelligent diagnostics of prognostic models” (see: the Acknowledgements section). The first research on the IPA, carried out in 2019–2020 (stage 1), made a significant contribution to the construction of the module supporting the construction and validation of various types of classification models. These studies were focused on analysing the effectiveness of various classification models in supporting credit decisions. Contribution included creation of decision models using seven different binary classifiers, five feature selection methods, as well as two data resampling and two feature discretization methods. Taking into account the number of methodological approaches considered in each group, this gave 315 different scenarios and the same number of classification models supporting credit decisions, which we evaluated. The research description and results were published in the article [9]. The dataset on which the experiment was conducted describes anonymized data about loan repayment and borrowers. This set consists of 91,759 records described by 272 conditional attributes (features) and the decision attribute. It was divided in proportion 70/30% into training set (64,230 records) and testing set (27,529 records). Both datasets are attached to article [9] (stored in the Journal repository). These data were also used to build on the IPA of various classification models that are the subject of research in this study.

In the next study, carried out in 2020 (stage 2), we focused on analysing the phenomenon of dataset shift and developing a systematic approach using a unified quantitative measure to continuously monitor classification models. The issue of dataset shift was so important that after a few or several months from the implementation of a fully operational predictive model at the client’s, it often turned out that the multidimensional distribution of data on which the model was created significantly differed from the incoming new data, which resulted in incorrect operation of this model at the client’s (increased risk of incorrect predictions).

The results and conclusions of the stage 1 and 2 studies influenced the final form of the IPA, which collects data for: modelling, monitoring and managing the life cycle of classification and forecasting models. The automated process of building classification models allows the analyst (expert) to generate many of their variants for different sets of explanatory variables and different parameter values required for each type of classifier. The IPA enables the construction of models based on:

- Logistic regression.
- Logistic regression with regularization.
- Random forest.
- XGBoost.

Note that Random Forest and eXtreme Gradient Boosting (XGBoost) are collaborative machine learning (decision tree based) methods. The procedure of building models using both of these methods requires slightly less involvement on the part of the analyst compared to the procedure based on logistic regression.

In the course of using the prototype version of IPA, its users encountered a problem, the solution of which required further research (stage 3) which is the subject of this study. The problem was that, on the one hand, the IPA offers the ease of building many variants of classification models, and on the other, as their number grows, the problem of evaluating and choosing the best model that would meet the requirements and preferences of the client (model user) to the highest degree increases.

The decision problem consisting in the assessment and selection of a classification model is multi-criteria in nature. When assessing this type of models, a number of measures (criteria) that define: strength, effectiveness and stability of the model should be taken into account. In the solution presented in the article, the selection of relevant measures for the study, from among all measures available on the IPA, was made by experts of BD Poland (creators and owners of IPA). They have over 10 years of experience in three areas: Financial Risk Management, Data Science and Artificial Intelligence Technology/They have built and implemented over 1,000 predictive models, a significant part of which are classification models for the purposes of credit scoring [62]. Five indicators were selected:

- *Gini*—a measure of model quality that can be interpreted as a percentage of the “ideal” of a given predictive model. The Gini coefficient is the area between the ROC curve (Receiver Operating Characteristic) [63] for the tested model and the ROC curve for the random model in percentage interpretation up to the value of 1/2—that is the area for the theoretically ideal classifier. It is a metric that evaluates the response of the model after it has been optimized.
- *Accuracy*—calculated as the quotient of the number of correctly classified cases to the entire set of cases (training or validation).
- *Precision*—precision of classification within the recognized class. It is calculated as the ratio of correctly classified elements from a given class (True Positive—TP) to all that the classifier has marked as this class (TP + FP; where FP—False Positive).
- *Recall*—understood as the number of objects of a given class recognized by the classifier. It is calculated as the ratio of correctly recognized elements from a given class (TP) to all that the classifier should recognize within the whole class (TP + FN; where FN—False Negative).
- F1 score—a measure of balance rating between *recall* a *precision*. This measure does not take into account true negatives (TN). It is calculated as the harmonic mean from the precision and sensitivity: F1 = (2 * precision * recall) /(precision + recall).

It should be emphasized that BD Poland experts use the above-mentioned indicators in their daily practice to evaluate predictive models, but in the case of a large number of their different variants generated on the IPA platform, it became very laborious and began to take much more time. There was a need to develop a tool dedicated to the IPA platform, which would systematically support the work of analysts in the assessment of predictive models. It was assumed that the above-mentioned indicators as criteria for evaluating predictive models will create a hierarchical structure which levels will be appropriately aggregated. Then, on the basis of the values of the criteria and the weights for these criteria declared by the expert, a ranking of these models will be prepared in a fully automated manner.

BD Poland experts submitted the values of the above-mentioned indicators for the study of 10 different classification models, which were created on the IPA platform using anonymized data on loan repayment and borrowers (see: collections from stage 1, attached to the article [9]). Based on their own experience and analysis of the proposed indicators, the experts evaluated and ranked the models in order to compare them with the ranking results obtained in the MCDA-based framework.

The value of each criterion was calculated twice, separately on the training and validation set, which gave a total of 10 sub-criteria for model evaluation. Additionally, all measure values were recorded on quarterly data—from the 1st quarter of 2017 (2017q1) to the 2nd quarter of 2019 (2019q2). Therefore, the decision problem was defined in three different dimensions, and in each of these dimensions it was described using several variables:

- Two variables for the sub-criteria dimension—training set, validation set,
- Five variables for the criteria dimension—GINI, accuracy, precision, recall, f1 score,
- Ten variables for the dimension of periods (quarters)—2017q1, 2017q2, …, 2019q2.

The structure of the criteria, sub-criteria and time periods is presented in Table 2.

**Table 2:**
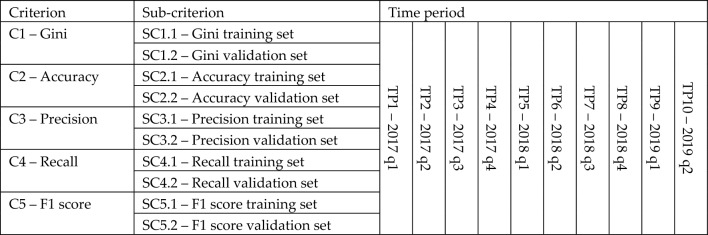
Structure of the criteria, sub-criteria and time periods used in the assessment of classifiers

Data for the study, in the form of classification results for 10 classification models (decision alternatives), were provided by a team of BD Poland experts preparing IPA (data are included in Appendix 1 and Table 3). The considered classification models (decision alternatives) and their number are summarized in Table 4. A total of 10 classifiers were assessed, including: 6 classification models based on logistic regression (A1, …, A6), 1—logistic regression with regularization (A7), 1—random forest method (A8) and 2—using the XGBoost method (A9, A10).

**Table 3:** The values of classifiers assessment indicators in the 2019q2 period

	C1		C2		C3		C4		C5	
	SC1.1	SC1.2	SC2.1	SC2.2	SC3.1	SC3.2	SC4.1	SC4.2	SC5.1	SC5.2
A1	0.5500	0.8100	0.6957	0.8261	0.0476	0.3333	0.5000	1.0000	0.0870	0.5000
A2	0.8200	0.8600	0.6522	0.7826	0.0769	0.2857	1.0000	1.0000	0.1429	0.4444
A3	0.5100	0.9000	0.7681	0.7826	0.0625	0.2857	0.5000	1.0000	0.1111	0.4444
A4	0.6400	0.9000	0.8551	0.8261	0.1000	0.3333	0.5000	1.0000	0.1667	0.5000
A5	0.5000	0.9000	0.8116	0.8261	0.0769	0.3333	0.5000	1.0000	0.1333	0.5000
A6	0.3900	1.0000	0.9710	0.3913	0.0000	0.1250	0.0000	1.0000	0.0000	0.2222
A7	0.8500	0.8100	0.6812	0.8261	0.0833	0.3333	1.0000	1.0000	0.1538	0.5000
A8	0.3600	0.7400	0.5072	0.6087	0.0294	0.1818	0.5000	1.0000	0.0556	0.3077
A9	0.2800	0.8600	0.9855	0.8696	1.0000	0.3333	0.5000	0.5000	0.6667	0.4000
A10	0.5700	0.9000	0.9855	0.9130	1.0000	0.5000	0.5000	0.5000	0.6667	0.5000

**Table 4:** Assessment classification models (decision alternatives)

No	Classification model	Number of predictive variables
A1	Logistic regression	7
A2	Logistic regression	6
A3	Logistic regression	6
A4	Logistic regression	5
A5	Logistic regression	5
A6	Logistic regression	5
A7	Logistic regression with regularization	23
A8	Random forest	44
A9	XGBoost	25
A10	XGBoost	28

According to BD Poland experts, it was important that the developed framework implement the following postulates.

- It should take into account the compatibility of the classification results with the use of the training set and the validation set (stability of the classification results regardless of the set of classified cases).
- It should prefer classifiers that give similar classification results for cases from different periods (stability of classification results over time).
- It should consider the quantitative parameters of classification results (criteria) as Gini measure, accuracy, precision, recall, and F1 score.

The last given requirement determines the use of multi-criteria methods in the assessment of classifiers. The fulfilment of the other two requirements is ensured by the use of the multi-criteria method called PROSA-C for the aggregation of the results of the training and validation sets, as well as for the aggregation of subsequent time periods. The PROSA-C method takes into account convergence between different variables. It can measure inconsistencies between classification results from different time periods or from different data sets (training and validation sets) and take these inconsistencies into account in the final evaluation of classifiers. Therefore, when aggregating sub-criteria (classification results for the training and validation set) and time periods, the PROSA-C method was used [29, 64]. At the stage of aggregation of criteria, such measurement of inconsistency was not required, therefore, when aggregating the criteria, the PROMETHEE II method, on which PROSA-C is based, was used.

### Methodological framework and applied PROMETHEE II and PROSA-C methods

**Fig. 1:**
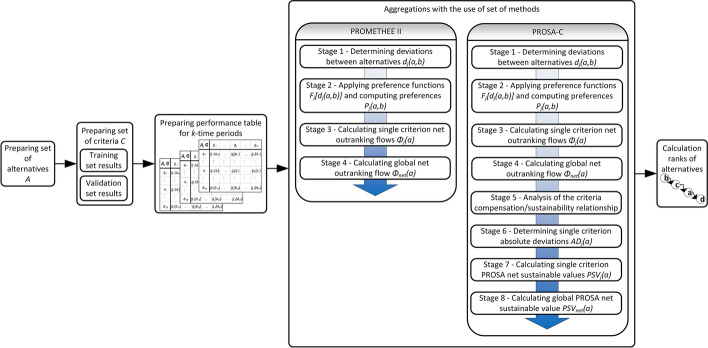
Framework for assessing classification models based on multiple criteria

The PROSA-C and PROMETHEE II methods were used in the framework shown in Fig. 1.

The PROSA-C method is used for examining discrete decision problems, where the set $A=\left\{a,b,\dots ,m\right\}$ with $M$ alternatives is considered. The alternatives are considered in terms of $n$ criteria belonging to the set $C=\left\{{c}_{1},{c}_{2},\dots ,{c}_{n}\right\}$. The PROSA-C method consists of 8 stages [65], with the initial 4 stages taken directly from the PROMETHEE II method, based on the single criterion net flows [66].

1. Determining the deviations based on pairwise comparisons.
2. Application of the preference functions.
3. Calculation of outranking flows for individual criteria.
4. Calculation of global net outranking flows.
5. Analysis of the balance/compensation criteria relationship.
6. Determination of absolute deviations for individual criteria.
7. Calculation of PROSA values for individual criteria.
8. Calculation of global PROSA-C values.

Stage 1. Determination of deviations based on pairwise comparisons.

In this step, all alternatives from the set $A$ are compared in pairs in terms of successive criteria ${c}_{j}$ and for each comparison the deviation ${d}_{j}$ is determined, according to the formula (1):

1 $${d}_{j}\left(a,b\right)={c}_{j}\left(a\right)-{c}_{j}\left(b\right), \forall a,b\in A, \forall j=1,\dots ,n,$$

where ${c}_{j}\left(a\right)$ is the rating/performance of the alternative $a$ for criterion ${c}_{j}$.

Stage 2. Application of the preference function.

For each *j*-th criterion, preference functions ${F}_{j}$ are selected, allowing the conversion of the deviation ${d}_{j}$ to the normalized preference value ${P}_{j}\in \left[\mathrm{0,1}\right]$, according to the formula (2):

2 $${P}_{j}\left(a,b\right)={F}_{j}\left[{d}_{j}\left(a,b\right)\right], \forall a,b\in A, \forall j=1,\dots ,n.$$

**Fig. 2:**
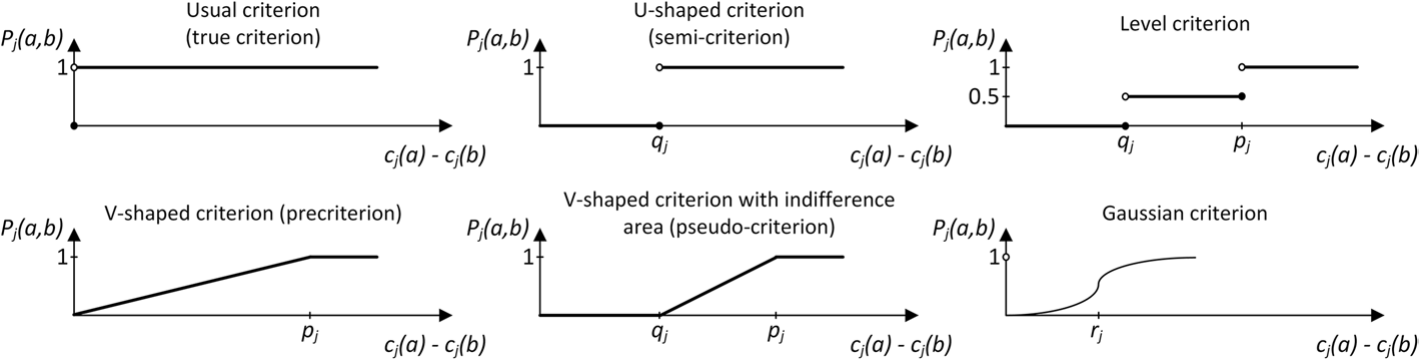
Preference functions used in the PROMETHEE and PROSA methods

At this stage, six different preference functions as shown in Fig. 2 can be applied.

These functions are described by the formulas (3, 4, 5, 6, 7 ,8), while in selected functions the following thresholds are used: ${q}_{j}$—indifference, ${p}_{j}$—preference, ${r}_{j}$—Gaussian.

- Usual criterion (true criterion) (3):
  3 $${P}_{j}\left(a,b\right)=\left\{\begin{array}{c}0\quad\mathrm{ for }\,{d}_{j}\left(a,b\right)\le 0 \\ 1\quad\mathrm{ for }\,{d}_{j}\left(a,b\right)>0.\end{array}\right.$$
- U-shaped criterion (semi-criterion) (4):
  4 $${P}_{j}\left(a,b\right)=\left\{\begin{array}{c}0\quad\mathrm{ for }\,{d}_{j}\left(a,b\right)\le {q}_{j} \\ 1\quad\mathrm{ for }\,{d}_{j}\left(a,b\right)>{q}_{j.}\end{array}\right.$$
- V-shaped criterion (pre-criterion) (5):
  5 $${P}_{j}\left(a,b\right)=\left\{\begin{array}{l}0\quad\mathrm{ for }\,{d}_{j}\left(a,b\right)\le 0\\ \frac{{d}_{j}\left(a,b\right)}{{p}_{j}}\quad\mathrm{ for }\,0\le{d}_{j}\left(a,b\right)\le {p}_{j}\\ 1\quad\mathrm{ for }\, 0<{d}_{j}\left(a,b\right)\le{p}_{j}.\end{array}\right.$$
- Level criterion (6):
  6 $${P}_{j}\left(a,b\right)=\left\{\begin{array}{l}0\quad\mathrm{ for }\,{d}_{j}\left(a,b\right)\le {q}_{j}\\ \frac{1}{2}\quad\mathrm{ for }\,{q}_{j}\le{d}_{j}\left(a,b\right)\le {p}_{j}\\ 1\quad\mathrm{ for }\,{d}_{j}\left(a,b\right)>{p}_{j}.\end{array}\right.$$
- V-shaped criterion with the area of indifference (pseudo-criterion) (7):
  7 $${P_j}\left( {a,b} \right) = \left\{ {\begin{array}{*{20}{c}} 0&{{\rm{for}}\,{d_j}\left( {a,b} \right) \le {q_j}}\\ {\frac{{{d_j}\left( {a,b} \right) - {q_j}}}{{{p_j} - {q_j}}}}&{{\rm{for}}\,{d_j} < \left( {a,b} \right) \le {p_j}\,}\\ 1&{{\rm{for}}\,{d_j} < \left( {a,b} \right) > {p_j}.} \end{array}} \right.$$
- Gaussian criterion (8):
  8 $${P_j}\left( {a,b} \right) = \left\{ {\begin{array}{*{20}{c}} 0&{{\text{for}}\,{d_j}\left( {a,b} \right) \le 0} \\ {1 - {\text{exp}}\left( {\frac{{ - {d_j}{{\left( {a,b} \right)}^2}}}{{2{r_j}^2}}} \right)}&{{\text{for}}\,{d_j}\left( {a,b} \right) > 0} \end{array}} \right.$$

Stage 3. Calculation of outranking flows for individual criteria.

Based on the preference value ${P}_{j}$, the outranking flow is calculated for each alternative in terms of each criterion, using the formula (9):

9 $${\phi }_{j}\left(a\right)=\frac{1}{M-1}\sum_{i=1}^{M}\left[{P}_{j}\left(a,{b}_{i}\right)-{P}_{j}\left({b}_{i},a\right)\right], \quad\forall a,{b}_{i}\in A, \forall j=1,\dots ,n,$$

where ${\phi }_{j}\left(a\right)$ is the alternative outranking flow $a$ over any other alternative for the *j*-th criterion, and $M$ is the number of alternatives. The values of ${\phi }_{j}$ allow the alternatives to be ordered separately for each criterion.

Stage 4. Calculation of the global net outranking flow.

The global net outranking flow for each of the alternative is determined on the basis of the formula (10):

10 $${\phi }_{net}\left(a\right)=\sum_{j=1}^{n}{\phi }_{j}\left(a\right){ w}_{j}, \quad\forall a\in A,$$

where ${w}_{j}$ is the weights of the *j*-th criterion, where the weights are normalized ($\sum_{j=1}^{n}{w}_{j}=1$). The standardization of weights is carried out in accordance with the formula (11):

11 $${w}_{j}=\frac{{ w}_{j}}{\sum_{j=1}^{n}{ w}_{j}} ,\quad\forall j=1,\dots ,n.$$

The obtained values of ${\phi }_{net}$ are the final solution for the application of the PROMETHEE II method. These four steps are performed in both the PROSA-C method and PROMETHEE. PROSA-C extends the PROMETHEE methodology with steps 5–8.

Stage 5. Analysis of the balance/criteria compensation relationship.

Once the values of ${\phi }_{net}\left(a\right)\mathrm\,{ and }\,{\phi }_{j}\left(a\right)$ have been determined, the decision-maker can examine whether the alternatives are sustainable on the basis of particular criteria. The PROSA methods distinguish between three balance/compensation relationships.

- The relation of being sustainable (balanced) (*≈*) – takes place when ${\phi }_{j}\left(a\right)\approx {\phi }_{net}\left(a\right)$ and means that the alternative *a* is sustainable in terms of the *j*-th criterion.
- The relation of being compensated (*Cd*) – occurs when ${\phi }_{j}\left(a\right)\ll {\phi }_{net}\left(a\right)$ and means that the low efficiency of criterion ${c}_{j}\left(a\right)$ is compensated by another criterion/criteria (${{\exists \phi }_{{j}^{^{\prime}}}\left(a\right):\phi }_{j}\left(a\right) Cd {\phi }_{{j}^{^{\prime}}}\left(a\right)$).
- Compensation relation (*Cs*) – occurs when ${\phi }_{j}\left(a\right)\gg {\phi }_{net}\left(a\right)$ and means that high performance of criterion ${c}_{j}\left(a\right)$ compensates lower performance on another criterion/criteria (${{\exists \phi }_{{j}^{^{\prime}}}\left(a\right):\phi }_{j}\left(a\right) Cs {\phi }_{{j}^{^{\prime}}}\left(a\right)$).

The *Cd* and *Cs* relations denoting the lack of balance of the alternative *a* in terms of the *j*-th criterion. The operators <  < and >  > denote the contractual relations “much less than” and “much greater than”. These relations express the subjective view of the decision maker as to whether the value on the left side of the operator is much smaller/much greater than the value on the right side, and therefore whether the alternative *a* is sustainable in terms of the *j*-th criterion or not. In turn, the operator $\approx$ means “approximately equal” and expresses the subjective view of the decision maker that the values on both sides of the operator can be considered equal. The analysis of the balance/compensation relationship can provide a clue for the decision-maker as to the expected values of the balance coefficients ${s}_{j}$. For example, if a decision maker wants to increase the impact of sustainability on the solution obtained, then a lower value of ${s}_{j}$ can be adopted for more sustainable criteria, and a higher value of ${s}_{j}$ for less sustainable criteria.

Stage 6. Determination of absolute deviations for individual criteria.

The values of absolute deviation are determined separately for each criteria, in accordance with the formula (12):

12 $${AD}_{j}\left(a\right)=\left|{\phi }_{net}\left(a\right)-{\phi }_{j}\left(a\right)\right|{s}_{j} , \quad \forall a\in A, \forall j=1,\dots ,n,$$

where ${s}_{j}$ is the balance (compensation) coefficient for the *j*-th criterion. It can be seen that ${s}_{j}$ is a kind of weighting factor, and ${AD}_{j}\left(a\right)$ is the weighted distance of the global solution ${\phi }_{net}\left(a\right)$ from the single-criteria solution ${\phi }_{j}\left(a\right)$.

Stage 7. Calculation of the sustainable PROSA values for the individual criteria.

For each alternative in terms of each criterion, a PROSA sustainable value is calculated (13):

13 $${PSV}_{j}\left(a\right)={\phi }_{j}\left(a\right)-{AD}_{j}\left(a\right), \forall a\in A, \quad \forall j=1,\dots ,n,$$

where ${PSV}_{j}\left(a\right)$ describes the balance of alternative *a* in terms of the *j*-th criterion.

Stage 8. Calculation of global PROSA-C net sustainable values.

PROSA net sustainable value is determined using the formula (14):

14 $${PSV}_{net}\left(a\right)=\sum_{j=1}^{n}{PSV}_{j}\left(a\right){ w}_{j}, \quad \forall a\in A.$$

Based on the ${PSV}_{net}$ value, a ranking of alternatives is built, with higher values of ${PSV}_{net}$ indicating a better final score [67].

**Fig. 3:**
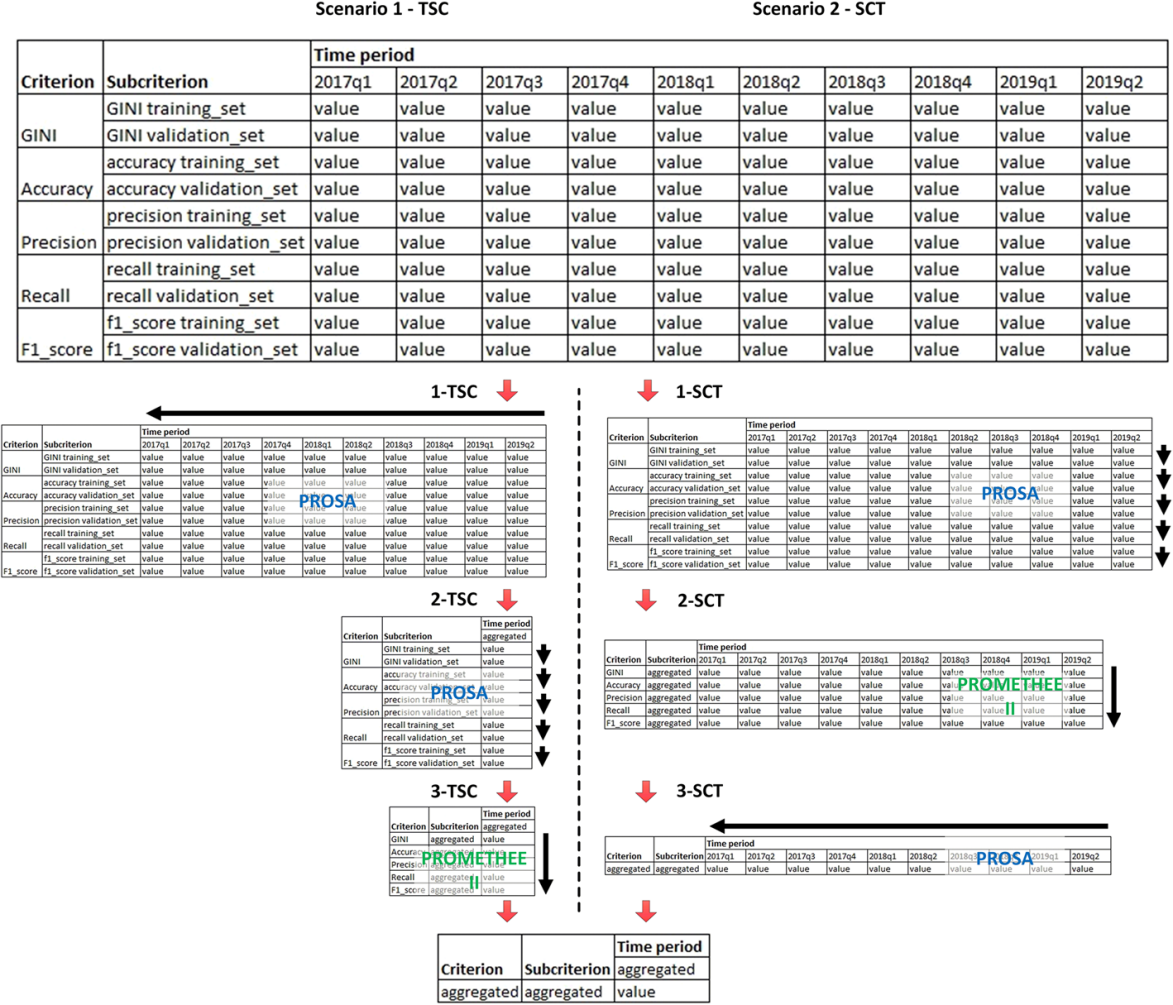
Scheme of successive aggregations in the developed framework

As part of solving the decision problem, for each of the alternative classifier models, the evaluation sub-criteria, criteria and periods of time in which a given sub-criterion reached a certain value were considered. The solution to the decision problem consisted in reducing all these values for a given alternative to a single synthesizing criterion. This was done by aggregating all variables in successive dimensions (sub-criteria, criteria and time periods). A diagram of subsequent aggregations in the developed framework is shown in Fig. 3.

The study considered two aggregation scenarios of TSC (Time periods, Sub-criteria, Criteria) and SCT (Sub-criteria, Criteria, Time periods). In the first scenario (TSC aggregation), initially, (1-TSC) the values of alternatives obtained for each of the sub-criteria in subsequent periods of time were aggregated into one alternative value for each sub-criterion. In this way, the time dimension of the classification results was eliminated. The next step (2-TSC) was to aggregate the values of the alternatives obtained for the two sub-criteria under the same criterion. In this way, the dimension of sub-criteria was eliminated. The last step (3-TSC) was to aggregate the criteria values into a single synthesizing criterion. In the second scenario (SCT aggregation), the aggregation order was changed, first (1-SCT) eliminating the sub-criteria dimension, then (2-SCT) aggregating to a single synthesizing criterion, and finally (3-SCT) eliminating the temporal dimension.

For different time periods and for both sub-criteria within the same criterion, the consistency (convergence) of the obtained results was important. It was important that the classifier allowed to obtain similar classification results on the training and validation set and in different time periods. Therefore, the PROSA-C method was used for the synthesis of time periods and the synthesis of sub-criteria, which allows to take into account the balance of these sub-criteria and time periods. On the other hand, the classic PROMETHEE II method was used to aggregate the criteria into a single synthesizing criterion.

## Results

### Multi-criteria decision model for the classifier evaluation

At the outset, the parameters of the preference model were obtained in the dialogue mode from the two experts in the form of proposed weights of individual sub-criteria, criteria and time periods. In the event of a difference of opinion, in the course of discussions, explanations and arguments of experts, efforts were made to reach a consensus (e.g. by proposing an average value of the weighting factor). Additionally, the directions and preference functions as well as the thresholds used in the PROMETHEE and PROSA methods were defined.

Very important in the PROMETHEE and PROSA methods is the selection of the appropriate preference function, which determines the uncertainty of the decision maker’s preferences in comparisons of pairs of alternatives. For qualitative criteria, it is recommended to use the usual criterion (true criterion), U-shaped criterion (semi-criterion), or level criterion. However, for quantitative criteria one of the following functions should be used: V-shaped criterion (pre-criterion), V-shaped criterion with the area of indifference (pseudo-criterion), or Gaussian criterion [68]. The simplest of them, and at the same time the easiest to interpret, is the pre-criterion, and this function was used in the preference model, because all the applied criteria for evaluating classification models are quantitative. According to Roy, the value of the preference threshold (${p}_{j}$) used in the pre-criterion should be between the reliable minimum and maximum values of a given criterion. Moreover, Roy points out that the values of the preference threshold can be based on characteristics describing a given criterion, e.g. mean, standard deviation, maximum, etc. [69]. Taking into account these recommendations, for the first stage of aggregation in each of the scenarios, the preference threshold *p* was set as the value of the sample standard deviation ${\sigma }_{jk}$ calculated from the value of a given *j*-th sub-criterion in a given *k*-th time period for *m* alternatives (${a}_{i} \bigwedge i=1\dots m$). As for the next stages of aggregation, the developed approach was modelled on the PROMETHEE GDSS method. In this method, the second aggregation step is based on the ${\phi }_{net}$ values obtained using the PROMETHEE II method in the first aggregation step. At this stage of the PROMETHEE GDSS method, a pre-criterion with a preference threshold of *p* = *2* is used [66]*.* Therefore, in the developed framework, the V-shaped criterion (pre-criterion) was used at the second and third stage of aggregation, and the preference threshold was *p* = *2*, which is the maximum possible difference between the result of the best (1) and worst (−1) alternative. In turn, the sustainability/compensation coefficient took the value ${s}_{jk}=0.5 \bigwedge j=1,\dots , n, k=1,\dots , t$). The preference model developed in this way is presented in Tables 5 and 6.

**Table 5:** The model of preferences in the problem of classifier assessment

Criterion	Weight	Sub-criterion	Weight	Time period	Weight	Preference direction	Preference function
C1–Gini	3	SC1.1—Training set	1	2017q1	1	Max	V-shaped
		SC1.2—Validation set	3	2017q2	1.05		
C2–Accuracy	1	SC2.1—Training set	1	2017q3	1.1		
		SC2.2—Validation set	3	2017q4	1.15		
C3–Precision	1	SC3.1—Training set	1	2018q1	1.2		
		SC3.2—Validation set	3	2018q2	1.25		
C4–Recall	1	SC4.1—Training set	1	2018q3	1.3		
		SC4.2—Validation set	3	2018q4	1.35		
C5–F1 score	1	SC5.1—Training set	1	2019q1	1.4		
		SC5.2—Validation set	3	2019q2	1.45

**Table 6:** Preference thresholds in the problem of classifier assessment

Preference threshold (*p*)—1st stage of aggregation	Preference threshold (*p*)—2nd stage of aggregation	Preference threshold (*p*)—3rd stage of aggregation
${\sigma }_{jk}=\sqrt{\frac{\sum_{i=1}^{m}{\left({c}_{jk}\left({a}_{i}\right)-\overline{{c }_{jk}\left(a\right)}\right)}^{2}}{m-1}}$	2	2

As already mentioned, the considered decision alternatives were the classifier models described by means of various assessment measures. These measures constituted sub-criteria and evaluation criteria. The complication was that individual measures were collected periodically, at quarterly intervals. Table 3 shows the most recent measures obtained for each classifier. These results came from the second quarter of 2019, while the results from the previous quarters are presented in Appendix 1.

The preference model along with the criteria values of alternatives created the so-called multi-criteria decision model, which is the basis for solving the decision problem and ordering the considered classifier models.

### Results of classifiers assessment using the TSC aggregation

The first stage of TSC (Time periods, Sub-criteria, Criteria) aggregation using the PROSA method consisted in reducing the time dimension to one value, taking into account the discrepancy between individual time periods. The weights of the individual time periods used during the aggregation, the directions of preferences, the preference functions and the values of the preference thresholds are presented in Tables 5 and 6. The ${PSV}_{net}$ values obtained for individual alternatives after the reduction of the time dimension are presented in Table 7.

**Table 7:** Criteria values of alternatives ${PSV}_{net}$ after aggregation of the time dimension in the TSC aggregation scheme

	C1		C2		C3		C4		C5	
	SC1.1	SC1.2	SC2.1	SC2.2	SC3.1	SC3.2	SC4.1	SC4.2	SC5.1	SC5.2
A1	− 0.0882	− 0.3749	− 0.3772	− 0.0629	− 0.3373	− 0.1799	0.0928	− 0.1553	− 0.3056	− 0.1575
A2	− 0.0347	0.1056	− 0.7702	− 0.3362	− 0.3969	− 0.1376	0.3509	0.1758	− 0.4014	− 0.1078
A3	− 0.0797	0.1373	− 0.0454	0.0875	− 0.2380	0.0704	0.0334	− 0.0548	− 0.0919	0.0873
A4	− 0.3387	0.0639	0.1185	0.2922	− 0.1994	0.2833	− 0.0747	− 0.1054	− 0.0047	0.3144
A5	− 0.2179	− 0.0033	− 0.1475	0.0974	− 0.2690	0.1314	0.1009	− 0.0548	− 0.1380	0.1616
A6	− 0.3635	− 0.5864	0.7374	− 0.9999	− 0.0117	− 0.5749	− 0.9824	0.3322	− 0.6946	− 0.6274
A7	− 0.3928	− 0.3430	− 0.3851	− 0.0911	− 0.3344	− 0.2748	0.3127	− 0.1837	− 0.2470	− 0.2665
A8	− 0.5314	− 0.6328	− 0.9406	− 0.7443	− 0.4347	− 0.4371	0.4667	0.2231	− 0.4962	− 0.4653
A9	0.0028	− 0.3837	0.7701	0.5333	0.7916	− 0.1674	− 0.4198	− 0.6823	0.9122	− 0.2837
A10	− 0.1243	− 0.0094	0.7693	0.7444	0.8287	− 0.5049	− 0.7848	− 0.8644	0.4557	− 0.5528

In the next step, sub-criteria were aggregated under each criterion. This simplified the decision problem into five criteria describing each of the ten alternatives. This aggregation was done taking into account the discrepancy between the sub-criteria within a given criterion, so it was carried out using the PROSA method. The sub-criterion weights, preference directions and preference functions are presented in Table 5. Regarding the preference threshold values, the *p* = *2* threshold was applied, as shown in Table 6. The ${PSV}_{net}$ values obtained after aggregating the sub-criteria are presented in Table 8.

**Table 8:** Criterial values of alternatives ${PSV}_{net}$ after aggregation of the sub-criteria dimension in the TSC aggregation scheme

	C1	C2	C3	C4	C5
A1	− 0.0852	− 0.0898	− 0.0676	− 0.0032	− 0.0396
A2	0.1406	− 0.2707	− 0.0689	0.1782	− 0.0474
A3	0.1396	0.0379	0.0348	0.0471	0.0888
A4	0.0537	0.1417	0.1107	0.0137	0.1810
A5	0.0620	0.0161	0.0463	0.0494	0.1009
A6	− 0.2049	− 0.4693	− 0.2045	− 0.0702	− 0.2810
A7	− 0.0866	− 0.1005	− 0.0966	− 0.0103	− 0.0594
A8	− 0.2349	− 0.4396	− 0.1717	0.2068	− 0.1821
A9	− 0.0867	0.3304	0.0357	− 0.2955	− 0.0138
A10	0.0829	0.4404	− 0.1388	− 0.4030	− 0.1698

The last aggregation concerned criteria and made it possible to obtain a general ranking of alternatives, and thus the ranking of classifier models. Similarly to the aggregation of the sub-criteria, the directions of preferences, preference functions and criteria weights, presented in Table 5, were used here. Also, as in the case of the sub-criteria, the value of *p* = *2* was adopted as the preference threshold. In this aggregation, the consistency of the criteria values was not taken into account therefore the aggregation was performed using the PROMETHEE II method. The final values of ${\phi }_{net}$ of alternatives and their ranking are presented in Table 9.

**Table 9:** Ranking of alternatives obtained from the TSC aggregation

Alternative	A1	A2	A3	A4	A5	A6	A7	A8	A9	A10
${\phi }_{net}$	− 0.0180	0.0351	0.0680	0.0665	0.0498	− 0.1119	− 0.0236	− 0.0843	0.0021	0.0164
Ranking	7	4	1	2	3	10	8	9	6	5

The analysis of Table 9 shows that the best classification results were achieved by classifier models based on logistic regression. They took 4 highest positions in the ranking, and the next two positions were taken by models using the XGBoost classifier. The last position in the ranking was taken by the classifier model, also based on logistic regression. This means that the quality of the classification was influenced not only by the classifier type used, but also by its parameters (defined in a given classification model). Comparing the number of variables used in individual classifier models, it can be concluded that the greater number of variables did not improve the quality of the classification model. The leading positions in the ranking were taken by models using a small number of variables, and classifiers using more than 20 variables took further positions.

### Results of classifiers assessment using the SCT aggregation

The first stage of SCT aggregation (Sub-criteria, Criteria, Time periods) was the aggregation of the sub-criteria dimension and obtaining the criteria scores separately for each of the time periods. This aggregation was performed using the PROSA method. The weights of individual sub-criteria, directions of preferences, preference functions and threshold values are presented in Tables 5 and 6. The values of ${PSV}_{net}$ obtained for individual alternatives after the reduction of the sub-criteria dimension are presented in Table 10.

**Table 10:** Criteria values of alternatives ${PSV}_{net}$ after aggregation of sub-criteria in the SCT aggregation scheme

	2017q1					2017q2				
	C1	C2	C3	C4	C5	C1	C2	C3	C4	C5
A1	− 0.2146	− 0.2184	− 0.0747	0.3290	0.0708	− 0.3053	− 0.3333	− 0.3374	− 0.5000	− 0.3926
A2	− 0.0105	− 0.6826	− 0.2672	0.2925	− 0.2003	0.1494	− 0.6096	− 0.1017	0.2083	− 0.1802
A3	0.0679	0.0638	0.1076	0.1727	0.2854	− 0.2208	0.0170	− 0.2618	− 0.5000	− 0.2201
A4	0.3496	0.1994	0.2185	0.1727	0.4509	0.0965	0.2771	− 0.1084	− 0.5000	0.0160
A5	− 0.0697	− 0.1210	0.0174	0.1727	0.1077	− 0.3411	− 0.0794	− 0.2904	− 0.5000	− 0.2866
A6	− 0.9227	− 1.2163	− 0.5255	− 0.5000	− 0.7025	− 1.0590	− 1.2183	− 0.6453	− 0.4167	− 0.5703
A7	− 0.9912	− 0.0490	− 0.7865	− 0.8845	− 0.8231	0.1586	− 0.1079	0.0497	0.5139	0.1183
A8	− 0.7296	− 0.7836	− 0.3074	0.2925	− 0.2664	− 0.9693	− 0.7935	− 0.2349	0.5139	− 0.2760
A9	0.3258	0.4848	− 0.9400	− 0.8479	− 0.9525	0.1573	0.5985	0.7775	0.2083	0.9684
A10	0.2395	0.8037	− 0.9211	− 0.7780	− 0.9232	− 0.3486	0.8301	− 1.2111	− 1.0278	− 1.2205

	2017q3					2017q4				
	C1	C2	C3	C4	C5	C1	C2	C3	C4	C5
A1	0.1118	− 0.2139	− 0.1364	0.0579	− 0.1192	− 0.2707	− 0.1969	− 0.2651	0.0748	− 0.0839
A2	0.0969	− 0.6943	− 0.3534	0.0579	− 0.3374	0.5537	− 0.5905	− 0.3966	0.0426	− 0.4547
A3	0.0969	0.1271	0.1590	0.0532	0.2138	− 0.4149	0.0898	− 0.0152	0.0748	0.2884
A4	− 0.1887	0.2221	0.1705	0.0532	0.2772	− 0.5317	0.2335	0.0488	-0.2629	0.1346
A5	0.0969	0.0346	0.0678	0.0532	0.1005	− 0.0270	0.1019	− 0.0377	0.0748	0.3000
A6	− 1.1298	− 1.2097	− 0.6362	− 0.5833	− 0.8436	− 0.7682	− 1.2212	− 0.7921	− 0.2500	− 0.8005
A7	0.1118	− 0.1630	− 0.1007	0.0579	− 0.0281	0.1018	− 0.2803	− 0.3538	0.1001	− 0.3262
A8	− 0.4707	− 0.8637	− 0.5582	0.0579	− 0.5492	− 1.1855	− 0.8754	− 0.6027	0.0426	− 0.7507
A9	− 0.5696	0.5508	0.8611	− 0.2515	0.9283	0.4225	0.3810	− 0.2072	− 0.9391	− 0.3851
A10	− 0.4410	0.7225	− 1.0991	− 1.0332	− 1.1268	− 0.3011	0.7276	1.0000	− 0.9198	0.8728

	2018q1					2018q2				
	C1	C2	C3	C4	C5	C1	C2	C3	C4	C5
A1	0.4030	− 0.3181	− 0.3056	0.3049	− 0.2585	0.0317	− 0.1911	− 0.1208	0.1333	− 0.0748
A2	− 0.2280	− 0.4460	− 0.2903	0.5431	− 0.1831	− 0.1932	− 0.4651	− 0.1583	0.1333	− 0.1467
A3	− 0.2280	0.0219	− 0.1983	− 0.6443	− 0.3303	0.1708	0.0547	0.0446	0.1333	0.1180
A4	− 0.8896	0.1934	− 0.1286	− 0.6443	− 0.2223	− 0.1799	0.1637	0.0765	− 0.7517	0.0860
A5	− 0.2531	0.0219	− 0.1924	− 0.6756	− 0.3591	− 0.3587	0.0891	0.2117	0.1333	0.2536
A6	− 0.9845	− 1.2181	− 0.4824	− 0.3895	− 0.8417	− 0.0889	− 1.2192	− 0.7811	− 0.5000	− 0.7500
A7	− 0.4269	− 0.2784	− 0.2917	0.3049	− 0.2272	0.5747	− 0.1887	− 0.1074	0.2738	− 0.0034
A8	− 0.0537	− 0.8951	− 0.4315	0.5431	− 0.5329	− 0.4731	− 0.8738	− 0.6793	0.2738	− 0.6636
A9	− 0.8184	0.7558	0.3544	− 0.6051	0.6460	− 1.1337	0.3413	0.7034	− 0.7517	0.3863
A10	0.5432	0.8540	1.0000	− 0.6721	0.8794	− 1.0292	0.4450	− 1.1637	− 1.0377	− 1.2008

	2018q3					2018q4				
	C1	C2	C3	C4	C5	C1	C2	C3	C4	C5
A1	− 0.6127	− 0.2904	− 0.6092	− 0.7990	− 0.6910	− 0.6224	− 0.1087	− 0.2406	− 0.0472	− 0.2185
A2	0.1228	− 0.5626	− 0.1752	0.2010	− 0.0160	0.3165	− 0.5442	− 0.3194	− 0.0472	− 0.3976
A3	0.2636	0.0882	0.1382	0.1998	0.4322	0.3220	0.0290	− 0.2001	− 0.0472	− 0.1033
A4	0.0220	0.2752	0.3560	0.4498	0.6779	− 0.1940	0.1651	− 0.1790	− 0.0056	− 0.1006
A5	− 0.1174	− 0.0588	0.0680	0.2010	0.3598	− 0.0983	− 0.1568	− 0.2516	− 0.0472	− 0.2449
A6	− 0.2714	− 1.2165	0.0485	− 0.2222	− 0.5128	− 0.9764	− 1.2204	− 0.3829	− 0.5556	− 0.5285
A7	− 0.6127	− 0.3097	− 0.5922	− 0.7733	− 0.6543	0.3220	− 0.2222	− 0.2621	− 0.0472	− 0.2605
A8	− 0.9095	− 0.7987	− 0.2757	0.1753	− 0.0979	− 0.5000	− 0.8414	− 0.3612	− 0.0472	− 0.4927
A9	− 0.7977	0.6948	− 0.7672	− 0.6806	− 0.8000	− 1.1250	0.6636	0.1168	− 0.3333	0.3748
A10	− 0.0151	0.7925	− 0.7394	− 0.8056	− 0.7697	0.1119	0.8180	0.7917	− 0.5556	0.7755

	2019q1					2019q2				
	C1	C2	C3	C4	C5	C1	C2	C3	C4	C5
A1	− 0.5432	− 0.1584	− 0.2928	− 0.4768	− 0.3548	− 0.6887	− 0.1922	− 0.0743	0.0139	− 0.0104
A2	− 0.0705	− 0.6654	− 0.2413	0.0942	− 0.3224	− 0.2148	− 0.3879	− 0.1720	0.1389	− 0.0721
A3	0.2356	0.0128	− 0.0020	0.0175	0.0637	0.0799	− 0.0993	− 0.1922	0.0139	− 0.1452
A4	0.1973	0.2741	0.2566	0.2446	0.4933	0.3453	0.1805	− 0.0011	0.0139	0.1730
A5	− 0.1374	− 0.0225	0.0081	0.0942	0.1207	0.0537	0.1082	− 0.0334	0.0139	0.0963
A6	− 0.4725	− 1.2159	− 0.1669	− 0.2811	− 0.0674	0.0058	− 1.2157	− 1.0265	− 0.5417	− 1.0340
A7	− 0.9839	− 0.3182	− 0.3430	− 0.5308	− 0.3669	− 0.7915	− 0.2242	− 0.0244	0.1389	0.1435
A8	− 0.7676	− 0.8154	− 0.1446	0.7214	− 0.1929	− 1.0357	− 0.9096	− 0.8929	0.0139	− 0.8327
A9	0.4396	0.7951	− 1.0972	− 0.9596	− 1.1250	− 0.5927	0.4018	0.1327	− 0.9861	− 0.6067
A10	− 0.0955	0.8186	− 1.1217	− 0.8774	− 1.0765	0.2279	0.6848	0.9306	− 0.9861	0.4077

In the next step, the criteria were aggregated using the PROMETHEE II method. In this way, 10 aggregated ratings for each alternative and 10 rankings were obtained, one for each time period. The applied directions of preferences, preference functions and weights of criteria are presented in Table 5. The value of *p* = *2* was adopted as the preference threshold. Assessments and rankings of alternatives obtained in particular time periods are presented in Table 11.

**Table 11:** Ratings and rankings of alternatives after criteria aggregation in the SCT aggregation scheme

	Aggregated ${\phi }_{net}$ values									
	2017q1	2017q2	2017q3	2017q4	2018q1	2018q2	2018q3	2018q4	2019q1	2019q2
A1	0.0796	− 0.0708	0.0966	0.0068	0.1648	0.1129	− 0.2018	− 0.0941	− 0.1056	− 0.0667
A2	0.0516	0.1073	0.0204	0.1294	0.0305	0.0290	0.1191	0.0744	0.0187	0.0279
A3	0.1883	− 0.0032	0.1696	0.0445	− 0.0310	0.1940	0.2646	0.1541	0.1889	0.1036
A4	0.2881	0.1239	0.1151	− 0.0058	− 0.1608	0.0489	0.2785	0.0472	0.2732	0.2294
A5	0.1196	− 0.0470	0.1460	0.1370	− 0.0412	0.0947	0.1510	0.0239	0.1087	0.1456
A6	− 0.3310	− 0.3520	− 0.4261	− 0.3175	− 0.3518	− 0.1536	− 0.0819	− 0.3425	− 0.1240	− 0.1835
A7	− 0.3156	0.2093	0.1107	0.0646	− 0.0261	0.2603	− 0.1971	0.1167	− 0.2325	− 0.0676
A8	− 0.1360	− 0.1675	− 0.1613	− 0.3472	− 0.0026	− 0.1414	− 0.1620	− 0.1544	− 0.0915	− 0.3365
A9	0.0208	0.3645	0.1323	0.1179	0.0112	− 0.0905	− 0.1795	− 0.0997	0.0407	− 0.1069
A10	0.0347	− 0.1646	− 0.2033	0.1703	0.4070	− 0.3542	0.0092	0.2744	− 0.0766	0.2547

	Rankings									
	2017q1	2017q2	2017q3	2017q4	2018q1	2018q2	2018q3	2018q4	2019q1	2019q2
A1	4	7	6	7	2	3	10	7	8	6
A2	5	4	7	3	3	6	4	4	5	5
A3	2	5	1	6	7	2	2	2	2	4
A4	1	3	4	8	9	5	1	5	1	2
A5	3	6	2	2	8	4	3	6	3	3
A6	10	10	10	9	10	9	6	10	9	9
A7	9	2	5	5	6	1	9	3	10	7
A8	8	9	8	10	5	8	7	9	7	10
A9	7	1	3	4	4	7	8	8	4	8
A10	6	8	9	1	1	10	5	1	6	1

The most recent reduction concerned time periods and allowed the classification of the classifier models in the aggregated rankings. In this case, the directions of preferences, the preference function and the weight of the periods of time presented in Table 5 and the preference threshold *p* = *2* were also used. The final values of ${PSV}_{net}$ alternatives and their ranking are presented in Table 12.

**Table 12:** Criterial values of alternatives ${PSV}_{net}$ after aggregation of the time dimension in the SCT aggregation scheme

Alternative	A1	A2	A3	A4	A5	A6	A7	A8	A9	A10
${PSV}_{net}$	− 0.0358	0.0228	0.0510	0.0378	0.0306	− 0.1728	− 0.0494	− 0.1154	− 0.0258	− 0.0308
Ranking	7	4	1	2	3	10	8	9	5	6

The ranking presented in Table 12 is very close to the ranking obtained using the TSC aggregation scheme presented in Table 9. The only difference was in positions 5 and 6, where the alternatives A9 and A10 swapped places. Therefore, the obtained results can be considered stable and reliable, although it should be noted that the order of aggregation is important and may affect the final results.

## Discussion

### Comparison of the PROSA solution with an expert empirical ranking

In order to verify the developed framework and the aggregation results obtained, the empirical results were compared with the results presented in Sects. “Results of classifiers assessment using the TSC aggregation” and “Results of classifiers assessment using the SCT aggregation”. A decision game was also carried out, which consisted in adjusting the decision model in such a way as to obtain a ranking as close as possible to the empirical ranking. At the beginning, experts were asked to organize the considered alternatives, obtaining an empirical ranking. This ranking is presented in Table 13.

**Table 13:** Empirical ranking of alternatives

Alternative	A1	A2	A3	A4	A5	A6	A7	A8	A9	A10
Ranking	7	1	2	4	5	10	8	9	6	3

In the next step, the decision model was adjusted in such a way as to obtain results as close to the given ranking as possible using TSC aggregation. Asa result of the conducted tests, it turned out that it is possible to obtain a ranking very similar to the ranking in Table 13 by manipulating only the weights of criteria and sub-criteria. In practice, all criteria had to be eliminated, except for C1—Gini, and the weight of its sub-criteria should be set to 1 for SC1.1—Training set and 2 for SC1.2—Validation set, respectively. In this case, the functions of preferences and time periods weights presented in Table 5 and the values of the preference thresholds presented in Table 6 remained unchanged. The ranking obtained in this way is presented in Table 14.

**Table 14:** Ranking obtained in the aggregation of TSC using only the C1 criterion

Alternative	A1	A2	A3	A4	A5	A6	A7	A8	A9	A10
${\phi }_{net}$	− 0.0284	0.0880	0.0846	0.0301	0.0416	− 0.0958	− 0.0350	− 0.1143	− 0.0277	0.0568
Ranking	7	1	2	5	4	9	8	10	6	3

The results presented in Table 14 allow for a thesis that the experts, when arranging the classifier models in the ranking, in practice based only on the C1—Gini criterion. This is confirmed by the fact that with the additional modification of the preference thresholds (*p*), it was possible to obtain a ranking exactly the same as empirical ranking presented in Table 13. The ranking together with the values of ${\phi }_{net}$ are presented in Table 15. The preference model used in this case is presented in Tables 16 and 17.

**Table 15:** Ranking obtained in the aggregation of TSC using the modified preference model

Alternative	A1	A2	A3	A4	A5	A6	A7	A8	A9	A10
${\phi }_{net}$	0.0017	0.0107	0.0093	0.0085	0.0057	− 0.0218	− 0.0115	− 0.0151	0.0038	0.0087
Ranking	7	1	2	4	5	10	8	9	6	3

**Table 16:** Preference model allowing for the order of alternatives to be the same as in the empirical ranking

Criterion	Weight	Sub-criterion	Weight	Time period	Weight	Preference direction	Preference function
C1-Gini	1	SC1.1—Training set	1	2017q1	1	Max	V-shaped
		SC1.2—Validation set	2	2017q2	1.05		
C2-Accuracy	0	SC2.1—Training set	0	2017q3	1.1		
		SC2.2—Validation set	0	2017q4	1.15		
C3-Precision	0	SC3.1—Training set	0	2018q1	1.2		
		SC3.2—Validation set	0	2018q2	1.25		
C4-Recall	0	SC4.1—Training set	0	2018q3	1.3		
		SC4.2—Validation set	0	2018q4	1.35		
C5-F1 score	0	SC5.1—Training set	0	2019q1	1.4		
		SC5.2—Validation set	0	2019q2	1.45

**Table 17:** Preference thresholds to obtain the same order of alternatives as in the empirical ranking

1st stage of aggregation		2nd stage of aggregation		3rd stage of aggregation	
Time period	Preference threshold (*p*)	Sub-criterion	Preference threshold (*p*)	Criterion	Preference threshold (*p*)
2017q1	2	SC1.1—Training set	2	C1—Gini	2
2017q2	2	SC1.2—Validation set	2		
2017q3	2	–	–	–	–
2017q4	2	–	–	–	–
2018q1	1.5	–	–	–	–
2018q2	2	–	–	–	–
2018q3	2	–	–	–	–
2018q4	2	–	–	–	–
2019q1	2	–	–	–	–
2019q2	2	–	–	–	–

### Comparison of the PROSA solution with solutions obtained using other MCDM methods

The results obtained using a framework based on the PROSA-C and PROMETHEE II methods were compared with the results of using other MCDM methods. The comparison included popular MCDM methods using quantitative data, i.e. SAW [70] and TOPSIS [71]. A variant of the framework was also considered, in which the PROMETHEE II method without the PROSA-C method was used in all stages of aggregation. In this study, the weights given in Table 5 were used. The results obtained by individual methods based on the TSC strategy are presented in Table 18. In addition, Table 19 re-quotes the results of the combination of PROSA-C and PROMETHEE II methods, as well as the empirical expert ranking.

**Table 18:** Rankings obtained in the TSC aggregation using the basic preference model and various MCDM methods

Method		A1	A2	A3	A4	A5	A6	A7	A8	A9	A10
Empirical ranking		7	1	2	4	5	10	8	9	6	3
PROSA-C + PROMETHEE II	${\phi }_{net}$	− 0.0180	0.0351	0.0680	0.0665	0.0498	− 0.1119	− 0.0236	− 0.0843	0.0021	0.0164
	Rank	7	4	1	2	3	10	8	9	6	5
PROMETHEE II	${\phi }_{net}$	− 0.0299	0.0109	0.0472	0.0657	0.0323	− 0.0842	− 0.0189	− 0.0917	0.0339	0.0347
	Rank	8	6	2	1	5	9	7	10	4	3
SAW	Score	77.61	82.55	88.74	96.72	86.80	65.10	75.07	69.70	87.60	84.10
	Rank	7	6	2	1	4	10	8	9	3	5
TOPSIS	Score	0.3701	0.6997	0.8361	0.9376	0.6201	0.2307	0.3446	0.2195	0.6854	0.7368
	Rank	7	4	2	1	6	9	8	10	5	3

**Table 19:** Correlation coefficients of rankings obtained in the TSC aggregation using the basic preference model

	PROSA-C + PROMETHEE II	PROMETHEE II	SAW	TOPSIS
Empirical ranking	0.7778	0.5556	0.5556	0.6889
PROSA-C + PROMETHEE II	–	0.6889	0.7778	0.7333
PROMETHEE II	–	–	0.8222	0.8667
SAW	–	–	–	0.7778

The mutual similarity of the rankings presented in Table 18 was examined using Kendall’s tau correlation, which is recommended for examining the convergence between the orders of alternatives [72]. The obtained correlation coefficients are presented in Table 19.

The correlation study showed that with the use of TSC aggregation, the developed framework based on the PROSA-C and PROMETHEE II methods allows to obtain the ranking of classifiers closest to the empirical expert ranking. The ranking obtained using the TOPSIS method is slightly less similar to the empirical ranking, and the PROMETHEE II and SAW rankings deviate the most from the empirical ranking.

In the same way, the results obtained with different MCDM methods using the SCT aggregation strategy were compared. These results are presented in Table 20.

**Table 20:** Rankings obtained in the SCT aggregation using the basic preference model and various MCDM methods

Method		A1	A2	A3	A4	A5	A6	A7	A8	A9	A10
Empirical ranking		7	1	2	4	5	10	8	9	6	3
PROSA-C + PROMETHEE II	${PSV}_{net}$	− 0.0358	0.0228	0.051	0.0378	0.0306	− 0.1728	− 0.0494	− 0.1154	− 0.0258	− 0.0308
	Rank	7	4	1	2	3	10	8	9	5	6
PROMETHEE II	${\phi }_{net}$	− 0.0299	0.0109	0.0472	0.0657	0.0323	− 0.0842	− 0.0189	− 0.0917	0.0339	0.0347
	Rank	8	6	2	1	5	9	7	10	4	3
SAW	Score	72.75	77.51	82.18	88.25	80.12	62.58	70.84	65.85	80.86	77.84
	Rank	7	6	2	1	4	10	8	9	3	5
TOPSIS	Score	0.5240	0.6741	0.7203	0.7176	0.6297	0.3509	0.5459	0.3479	0.5690	0.6428
	Rank	8	3	1	2	5	9	7	10	6	4

Kendall’s tau correlation coefficients for the compared rankings are presented in Table 21.

**Table 21:** Correlation coefficients of rankings obtained in the SCT aggregation using the basic preference model

	PROSA-C + PROMETHEE II	PROMETHEE II	SAW	TOPSIS
Empirical ranking	0.7333	0.5556	0.5556	0.7778
PROSA-C + PROMETHEE II	–	0.6444	0.8222	0.7778
PROMETHEE II	–	–	0.8222	0.7778
SAW	–	–	–	0.6000

In the case of SCT aggregation, the TOPSIS ranking shows the greatest similarity to the empirical ranking. A lower correlation with the empirical ranking shows the ranking obtained on the basis of combining the PROSA-C and PROMETHEE II methods. As with the TSC aggregation, the PROMETHEE II and SAW rankings differ the most from the empirical ranking.

When comparing the correlation coefficients of individual rankings with the empirical ranking, it should be noted that the TOPSIS ranking correlation coefficient in the SCT strategy has the same value as the PROSA-C + PROMETHEE II ranking correlation coefficient in the TSC strategy (0.7778). However, it was the PROSA-C + PROMETHEE II ranking that obtained the second highest correlation score with the empirical ranking (0.7333 in the SCT strategy), ahead of the TOPSIS ranking (0.6889 in the TSC strategy). This proves that with the assumed parameters of the decision model, the combination of the PROSA-C and PROMETHEE II methods gives the results of the assessment of classification algorithms closest to the empirical ranking, which was constructed by experts dealing with the issue of building classifiers for credit scoring purposes. This observation is confirmed by the search for such a decision model for the TOPSIS method, which would allow obtaining a ranking closest to the empirical ranking. It should be recalled here that in the case of combining the PROSA-C and PROMETHEE II methods, the ranking most similar to the empirical one was obtained using the TSC aggregation by applying only the C1 criterion with the weights of the sub-criteria SC1.1 = 1 and SC1.2 = 2, without changing other parameters of the decision model. Kendall’s tau correlation with the empirical ranking is 0.9111. In the case of the TOPSIS method, in order to maximize the correlation with the empirical ranking, the weights of the SC1.2 sub-criterion had to be changed from 3 to 4, leaving the other elements of the decision model unchanged. However, such a modification made it possible to obtain a correlation of only 0.8222. In addition, it should be noted that in the case of the decision model based on PROSA-C and PROMETHEE II, along with the improvement of the correlation of the results of this model with the empirical ranking, a significant simplification of the decision model itself was also obtained. In the revised decision model, 1 criterion with 2 sub-criteria and 10 time periods was left. Considering the complexity of the basic problem, which included 5 criteria, 10 sub-criteria and 10 time periods, this is a significant reduction in the complexity of the model. On the other hand, in the case of the model based on the TOPSIS method, no reduction in complexity was obtained. Another important benefit of using the approach based on the PROSA-C and PROMETHEE II methods is the flexibility of such a decision-making model, the possibility of various modifications and adapting it to the preferences of experts/decision makers. In the TOPSIS and SAW methods, you can only change the weights of the criteria/sub-criteria/time periods, while the PROSA and PROMETHEE II methods allow you to change the preference functions, thresholds, weights, compensation factor, etc. In the case under study, this allowed the decision model to be modified in such a way that it accurately reflects the empirical ranking of classification models developed by experts (see Sect. “Comparison of the PROSA solution with an expert empirical ranking”). In other words, the flexibility of the decision model allows it to be calibrated.

## Conclusion

The results of the study indicate that, regardless of the aggregation scenario adopted, the results of the assessment of classification models using the combination of the PROSA-C and PROMETHEE II methods were very similar. These results between the TSC and SCT aggregation strategies differ only for items 5 and 6. Based on the ranking of classifiers obtained from the experts, it was found that most likely they only use the C1 criterion, despite the fact that they declare that the other criteria are also important. Therefore, based on the preference model defined by field experts and using the PROSA/PROMETHEE set of methods, it was found that the best classification models are A3, A4, A5 and A2, and thus models based on logistic regression. On the other hand, the expert ranking indicated that the best classification models are A2, A3, A10 and A4. Therefore, this ranking is similar to the ranking obtained using the MCDM methods and the expert-defined preference model, except that the A10 alternative ranks high in the expert ranking. Based on the cited rankings, it should be stated that the leading positions are occupied by models of borrower classification, based on the classic approach using logistic regression and a small number of predictive variables. According to experts, the classification model based on XGBoost also achieves good results, but it uses a much larger number of predictor variables than the models based on logistic regression.

As for the scientific contribution, it should be noted that as a result of the conducted research, a systematic approach to multi-criteria evaluation and ranking of classification models has been developed. The approach developed is applicable not only to credit scoring classification problems, but can be generalized to any area of classification problems. The proposed approach uses the PROSA-C method. Thanks to this, the ranking of models takes into account the consistency of the model results in the sub-criteria and time dimensions. The results of comparative studies have shown that the PROSA-C method enables the ordering of classification models in a very similar order to the one established by experts, and with additional modification of the decision model it is possible to fully reflect the implicit preferences of the decision-maker. Comparing the results of the evaluation of classifiers using different MCDM methods (TOPSIS, SAW, PROMETHEE II, PROSA-C + PROMETHEE II), it should be stated that the developed framework combining the PROMETHEE II and PROSA-C methods allows to obtain the results of the evaluation most similar to the expert evaluation. Rankings created using other MCDM methods are less correlated with expert empirical ranking. Meanwhile, when evaluating classifiers, care should be taken to ensure a relatively high degree of compatibility of the mathematical evaluation model with the expert mental model, because otherwise the expert may avoid using the recommendations of the automated evaluation system and lose confidence in it [22].

Summing up the conclusions from the research, some general observations regarding the developed framework and the results of classifier evaluation can be specified.

- Classification models based on logistic regression, using a small number of predictor variables, received the highest scores.
- The order of aggregation of criteria, sub-criteria and time periods affects the result of evaluating classification models and their ranking.
- The developed decision model using a combination of PROSA-C and PROMETHEE II methods in the considered case gives results closest to the mental evaluation model, expressed in the form of an expert empirical ranking.
- Although the experts declared the use of as many as five criteria in the empirical assessment, matching the decision model to the mental model showed that in practice they used only the C1 criterion (Gini measure).
- The PROSA-C and PROMETHEE II methods used in the study, compared to other methods, make it possible to adjust the decision-making model to the experts’ preferences and their mental model.

Among the quoted conclusions, the basic advantage of the proposed framework can be indicated, which is the possibility of obtaining a decision model very close to the mental model of the expert/experts. Moreover, even if the decision model does not sufficiently reflect the mental model (the ranking obtained based on the framework differs from the empirical ranking), thanks to the numerous parameters used in the PROSA-C and PROMETHEE II methods, it is possible to relatively easily match the decision model to the mental model. In addition, the developed framework takes into account the compatibility of the classification results using the training set and the validation set, as well as the compatibility of the classification results for cases from different periods. Thanks to this, he prefers classification models that ensure the stability of the classification results regardless of the set of cases and the stability of the classification results over time. Another obvious advantage, which was also the purpose of developing the framework, is the ability to partially automate the assessment of classification models, which allows to redirect the efforts of experts to other areas of IPA operation. As for the imperfections and limitations of the proposed framework, the most important drawback relates to the need to calibrate the decision-making model so that it reflects the expert’s mental model. This can be a time-consuming process, and it can be assumed that even with the exact adjustment of parameters, the mathematical model of evaluation will not always fully reflect the mental model. In other words, the rankings of classifiers generated by the decision model may differ to some extent from expert empirical rankings, even despite attempts to adjust the decision model. This assumption is related to the research limitations of this study, because we tested the developed decision model based on the opinions and information taken from two experts. Probably, the involvement of more experts dealing with the issues of classification would result in an increase in the quality of the decision model and the recommendations generated by it.

The obtained research results do not close the issues related to multi-criteria evaluation of classification models. The problem of assessing classification models is so complex that it requires further research and experimental research. Future research will include, above all, taking into account additional evaluation criteria. In some cases, apart from the classification results, there may also be a significant number of predictive variables in the classification model, the “explainability” of the classifier, and thus the ease of explaining its decisions. Moreover, even taking into account only the results of the classification, it is impossible to clearly determine whether it is better to use the ROC curve and the Gini measure, or the PRC curve (the mean value under the curve, or calculated only for negative or only positive cases). Often, the selection of a classifier model is based on the search for a compromise between the various features of individual classification models. Another important research direction is the sensitivity analysis of the developed evaluation model. Performing sensitivity analysis is difficult because the decision problem is placed in three dimensions: criteria (e.g. Gini measure, accuracy, precision, recall, etc.), sub-criteria (e.g. classification results on training and validation sets), and periods (classification results on datasets from different time periods). Therefore, the sensitivity analysis would have to take into account changes in the weights of individual factors in each of these dimensions: a change in the weights of each criteria, a change in the weights of each of the sub-criteria, and a change in the weights of each of the time periods.

## Data Availability

Data are contained within the article.

## Appendix 1

See Tables 22, 23, 24, 25, 26, 27, 28, 29, 30

**Table 22:** Criterial values of alternatives in the 2017q1 time period

	C1		C2		C3		C4		C5	
	SC1.1	SC1.2	SC2.1	SC2.2	SC3.1	SC3.2	SC4.1	SC4.2	SC5.1	SC5.2
A1	0.7300	0.5600	0.8008	0.7679	0.1754	0.0714	0.8333	1.0000	0.2899	0.1333
A2	0.7100	0.6700	0.7276	0.6786	0.1429	0.0526	0.9167	1.0000	0.2472	0.1000
A3	0.7000	0.8500	0.8455	0.8214	0.2045	0.0909	0.7500	1.0000	0.3214	0.1667
A4	0.7200	0.9600	0.8699	0.8393	0.2368	0.1000	0.7500	1.0000	0.3600	0.1818
A5	0.7000	0.7100	0.8008	0.8214	0.1636	0.0909	0.7500	1.0000	0.2687	0.1667
A6	0.7800	0.2400	0.9512	0.2500	0.5000	0.0233	0.0833	1.0000	0.1429	0.0455
A7	0.6600	0.0200	0.8333	0.7857	0.1915	0.0000	0.7500	0.0000	0.3051	0.0000
A8	0.6600	0.3500	0.7114	0.6429	0.1358	0.0476	0.9167	1.0000	0.2366	0.0909
A9	0.8500	0.7800	0.9715	0.8929	0.7273	0.0000	0.6667	0.0000	0.6957	0.0000
A10	0.7300	0.7500	0.9593	0.9821	0.6250	0.0000	0.4167	0.0000	0.5000	0.0000

**Table 23:** Criterial values of alternatives in the 2017q2 time period

	C1		C2		C3		C4		C5	
	SC1.1	SC1.2	SC2.1	SC2.2	SC3.1	SC3.2	SC4.1	SC4.2	SC5.1	SC5.2
A1	0.6100	0.4400	0.7912	0.6753	0.0526	0.0400	0.5000	0.5000	0.0952	0.0741
A2	0.6500	0.6200	0.7253	0.6494	0.0400	0.0690	0.5000	1.0000	0.0741	0.1290
A3	0.6100	0.4900	0.8645	0.7143	0.0811	0.0455	0.5000	0.5000	0.1395	0.0833
A4	0.7400	0.6300	0.9011	0.7792	0.1111	0.0588	0.5000	0.5000	0.1818	0.1053
A5	0.6500	0.4500	0.8425	0.7013	0.0698	0.0435	0.5000	0.5000	0.1224	0.0800
A6	0.6300	0.0700	0.9817	0.2078	1.0000	0.0317	0.1667	1.0000	0.2857	0.0615
A7	0.6200	0.7700	0.8278	0.7143	0.0816	0.0833	0.6667	1.0000	0.1455	0.1538
A8	0.6500	0.1900	0.7143	0.5325	0.0500	0.0526	0.6667	1.0000	0.0930	0.1000
A9	0.6100	0.8700	0.9853	0.8442	0.7500	0.1429	0.5000	1.0000	0.6000	0.2500
A10	0.4600	0.8300	0.9853	0.9610	1.0000	0.0000	0.3333	0.0000	0.5000	0.0000

**Table 24:** Criterial values of alternatives in the 2017q3 time period

	C1		C2		C3		C4		C5	
	SC1.1	SC1.2	SC2.1	SC2.2	SC3.1	SC3.2	SC4.1	SC4.2	SC5.1	SC5.2
A1	0.9500	1.0000	0.8117	0.7963	0.1064	0.0833	1.0000	1.0000	0.1923	0.1538
A2	0.8900	1.0000	0.7130	0.7222	0.0725	0.0625	1.0000	1.0000	0.1351	0.1176
A3	0.8900	1.0000	0.8520	0.9074	0.1111	0.1667	0.8000	1.0000	0.1951	0.2857
A4	0.8500	1.0000	0.8834	0.8889	0.1379	0.1429	0.8000	1.0000	0.2353	0.2500
A5	0.8900	1.0000	0.8341	0.8889	0.1000	0.1429	0.8000	1.0000	0.1778	0.2500
A6	0.8900	0.3600	0.9731	0.4074	0.0000	0.0303	0.0000	1.0000	0.0000	0.0588
A7	0.9500	1.0000	0.8161	0.8148	0.1087	0.0909	1.0000	1.0000	0.1961	0.1667
A8	0.7900	1.0000	0.6816	0.5741	0.0658	0.0417	1.0000	1.0000	0.1235	0.0800
A9	0.7800	0.9600	0.9731	0.9444	0.4286	0.2500	0.6000	1.0000	0.5000	0.4000
A10	0.9100	0.8900	0.9821	0.9815	0.6667	0.0000	0.4000	0.0000	0.5000	0.0000

**Table 25:** Criterial values of alternatives in the 2017q4 time period

	C1		C2		C3		C4		C5	
	SC1.1	SC1.2	SC2.1	SC2.2	SC3.1	SC3.2	SC4.1	SC4.2	SC5.1	SC5.2
A1	0.6200	0.4500	0.7336	0.7586	0.1290	0.1333	0.7273	0.6667	0.2192	0.2222
A2	0.6500	0.5200	0.6542	0.6724	0.1111	0.1000	0.8182	0.6667	0.1957	0.1739
A3	0.6300	0.4400	0.7804	0.8448	0.1538	0.2000	0.7273	0.6667	0.2540	0.3077
A4	0.5700	0.4700	0.8037	0.8966	0.1220	0.2857	0.4545	0.6667	0.1923	0.4000
A5	0.6200	0.4700	0.7944	0.8276	0.1633	0.1818	0.7273	0.6667	0.2667	0.2857
A6	0.5600	0.4200	0.9439	0.2414	0.3333	0.0638	0.0909	1.0000	0.1429	0.1200
A7	0.6000	0.5600	0.7336	0.7069	0.1167	0.1111	0.6364	0.6667	0.1972	0.1905
A8	0.6300	0.3100	0.5607	0.5345	0.0891	0.0714	0.8182	0.6667	0.1607	0.1290
A9	0.6200	0.5500	0.9393	0.8621	0.4167	0.1429	0.4545	0.3333	0.4348	0.2000
A10	0.6000	0.4500	0.9579	0.9483	0.6667	0.5000	0.3636	0.3333	0.4706	0.4000

**Table 26:** Criterial values of alternatives in the 2018q1 time period

	C1		C2		C3		C4		C5	
	SC1.1	SC1.2	SC2.1	SC2.2	SC3.1	SC3.2	SC4.1	SC4.2	SC5.1	SC5.2
A1	0.6400	0.9000	0.7522	0.6863	0.1311	0.1579	0.6667	1.0000	0.2192	0.2727
A2	0.6000	0.7800	0.7261	0.6667	0.1408	0.1500	0.8333	1.0000	0.2410	0.2609
A3	0.6000	0.7800	0.8304	0.7059	0.1860	0.1250	0.6667	0.6667	0.2909	0.2105
A4	0.5900	0.6900	0.8565	0.7451	0.2162	0.1429	0.6667	0.6667	0.3265	0.2353
A5	0.6500	0.7600	0.8304	0.7059	0.2000	0.1250	0.7500	0.6667	0.3158	0.2105
A6	0.7400	0.7000	0.9435	0.2549	0.3333	0.0732	0.0833	1.0000	0.1333	0.1364
A7	0.5300	0.8300	0.7696	0.6667	0.1404	0.1500	0.6667	1.0000	0.2319	0.2609
A8	0.6100	0.8100	0.6391	0.4902	0.1099	0.1034	0.8333	1.0000	0.1942	0.1875
A9	0.7600	0.7200	0.9435	0.8824	0.4444	0.2857	0.3333	0.6667	0.3810	0.4000
A10	0.6800	0.8500	0.9565	0.9804	0.7500	1.0000	0.2500	0.6667	0.3750	0.8000

**Table 27:** Criterial values of alternatives in the 2018q2 time period

	C1		C2		C3		C4		C5	
	SC1.1	SC1.2	SC2.1	SC2.2	SC3.1	SC3.2	SC4.1	SC4.2	SC5.1	SC5.2
A1	0.6800	0.8800	0.7638	0.8491	0.0625	0.2727	0.6000	1.0000	0.1132	0.4286
A2	0.5600	0.9300	0.6935	0.8491	0.0484	0.2727	0.6000	1.0000	0.0896	0.4286
A3	0.6500	0.9100	0.8141	0.8868	0.0789	0.3333	0.6000	1.0000	0.1395	0.5000
A4	0.6300	0.8500	0.8392	0.9057	0.0909	0.3333	0.6000	0.6667	0.1579	0.4444
A5	0.5100	0.9300	0.8040	0.9245	0.0750	0.4286	0.6000	1.0000	0.1333	0.6000
A6	0.6100	0.8800	0.9648	0.3585	0.0000	0.0811	0.0000	1.0000	0.0000	0.1500
A7	0.7500	0.9500	0.7739	0.8302	0.0833	0.2500	0.8000	1.0000	0.1509	0.4000
A8	0.5200	0.8800	0.6482	0.6038	0.0548	0.1250	0.8000	1.0000	0.1026	0.2222
A9	0.6800	0.5900	0.9799	0.9245	0.6000	0.4000	0.6000	0.6667	0.6000	0.5000
A10	0.8500	0.6800	0.9749	0.9434	0.5000	0.0000	0.4000	0.0000	0.4444	0.0000

**Table 28:** Criterial values of alternatives in the 2018q3 time period

	C1		C2		C3		C4		C5	
	SC1.1	SC1.2	SC2.1	SC2.2	SC3.1	SC3.2	SC4.1	SC4.2	SC5.1	SC5.2
A1	0.6400	0.3300	0.7594	0.6852	0.1887	0.0000	0.8333	0.0000	0.3077	0.0000
A2	0.6800	0.5200	0.6952	0.6852	0.1538	0.0588	0.8333	0.5000	0.2597	0.1053
A3	0.6800	0.6500	0.8182	0.7778	0.2250	0.0833	0.7500	0.5000	0.3462	0.1429
A4	0.6400	0.7300	0.8289	0.8704	0.2368	0.2222	0.7500	1.0000	0.3600	0.3636
A5	0.6600	0.4700	0.7861	0.7593	0.2083	0.0769	0.8333	0.5000	0.3333	0.1333
A6	0.5600	0.8300	0.9251	0.2407	0.2500	0.0465	0.0833	1.0000	0.1250	0.0889
A7	0.6400	0.3300	0.7326	0.7593	0.1607	0.0000	0.7500	0.0000	0.2647	0.0000
A8	0.7300	0.2900	0.6738	0.5370	0.1549	0.0400	0.9167	0.5000	0.2651	0.0741
A9	0.7300	0.3200	0.9519	0.9259	0.7143	0.0000	0.4167	0.0000	0.5263	0.0000
A10	0.7400	0.4900	0.9358	0.9630	0.5000	0.0000	0.0833	0.0000	0.1429	0.0000

**Table 29:** Criterial values of alternatives in the 2018q4 time period

	C1		C2		C3		C4		C5	
	SC1.1	SC1.2	SC2.1	SC2.2	SC3.1	SC3.2	SC4.1	SC4.2	SC5.1	SC5.2
A1	0.7700	0.9700	0.7809	0.7705	0.1286	0.0667	0.9000	1.0000	0.2250	0.1250
A2	0.8000	1.0000	0.7173	0.6721	0.1023	0.0476	0.9000	1.0000	0.1837	0.0909
A3	0.7900	1.0000	0.8092	0.7869	0.1452	0.0714	0.9000	1.0000	0.2500	0.1333
A4	0.7400	1.0000	0.8163	0.8525	0.1379	0.1000	0.8000	1.0000	0.2353	0.1818
A5	0.7500	1.0000	0.7703	0.7705	0.1233	0.0667	0.9000	1.0000	0.2169	0.1250
A6	0.7600	0.9300	0.9647	0.3279	0.5000	0.0238	0.3000	1.0000	0.3750	0.0465
A7	0.7900	1.0000	0.7774	0.7049	0.1268	0.0526	0.9000	1.0000	0.2222	0.1000
A8	0.6700	1.0000	0.6784	0.5246	0.0909	0.0333	0.9000	1.0000	0.1651	0.0645
A9	0.8800	0.9300	0.9788	0.9180	0.7500	0.1667	0.6000	1.0000	0.6667	0.2857
A10	0.7700	1.0000	0.9647	1.0000	0.5000	1.0000	0.3000	1.0000	0.3750	1.0000

**Table 30:** Criterial values of alternatives in the 2019q1 time period

	C1		C2		C3		C4		C5	
	SC1.1	SC1.2	SC2.1	SC2.2	SC3.1	SC3.2	SC4.1	SC4.2	SC5.1	SC5.2
A1	0.7000	0.2900	0.7593	0.7091	0.0984	0.0667	0.6667	0.3333	0.1714	0.1111
A2	0.7300	0.4400	0.6722	0.5636	0.0833	0.0800	0.7778	0.6667	0.1505	0.1429
A3	0.7400	0.6000	0.8216	0.6909	0.1304	0.1111	0.6667	0.6667	0.2182	0.1905
A4	0.7300	0.6200	0.8714	0.7455	0.1765	0.1765	0.6667	1.0000	0.2791	0.3000
A5	0.7700	0.4200	0.8091	0.6909	0.1373	0.1111	0.7778	0.6667	0.2333	0.1905
A6	0.7600	0.3300	0.9627	0.2909	0.5000	0.0714	0.2222	1.0000	0.3077	0.1333
A7	0.6100	0.1200	0.7386	0.6545	0.1029	0.0556	0.7778	0.3333	0.1818	0.0952
A8	0.8200	0.2700	0.6432	0.4909	0.0860	0.0968	0.8889	1.0000	0.1569	0.1765
A9	0.9100	0.5600	0.9793	0.8727	0.7500	0.0000	0.6667	0.0000	0.7059	0.0000
A10	0.6800	0.6300	0.9710	0.9273	1.0000	0.0000	0.2222	0.0000	0.3636	0.0000
